# Brain extraction on MRI scans in presence of diffuse glioma: Multi-institutional performance evaluation of deep learning methods and robust modality-agnostic training^☆^

**DOI:** 10.1016/j.neuroimage.2020.117081

**Published:** 2020-06-27

**Authors:** Siddhesh Thakur, Jimit Doshi, Sarthak Pati, Saima Rathore, Chiharu Sako, Michel Bilello, Sung Min Ha, Gaurav Shukla, Adam Flanders, Aikaterini Kotrotsou, Mikhail Milchenko, Spencer Liem, Gregory S. Alexander, Joseph Lombardo, Joshua D. Palmer, Pamela LaMontagne, Arash Nazeri, Sanjay Talbar, Uday Kulkarni, Daniel Marcus, Rivka Colen, Christos Davatzikos, Guray Erus, Spyridon Bakas

**Affiliations:** a Center for Biomedical Image Computing and Analytics (CBICA), University of Pennsylvania, Philadelphia, PA, USA; b Shri Guru Gobind Singhji Institute of Engineering and Technology, Nanded, Maharashtra, India; c Department of Radiology, Perelman School of Medicine, University of Pennsylvania, Philadelphia, PA, USA; d Department of Radiation Oncology, Christiana Care Health System, Philadelphia, PA, USA; e Department of Radiology, Thomas Jefferson University, Philadelphia, PA, USA; f Department of Radiation Oncology, Sidney Kimmel Cancer Center, Thomas Jefferson University, Philadelphia, PA, USA; g Department of Diagnostic Radiology, University of Texas MD Anderson Cancer Center, TX, USA; h Department of Radiology, Washington University, School of Medicine, St. Louis, MO, USA; i Sidney Kimmel Medical College, Thomas Jefferson University, Philadelphia, PA, USA; j Department of Radiation Oncology, University of Maryland, Baltimore, MD, USA; k Department of Radiation Oncology, James Cancer Center, The Ohio State University, Columbus, OH, USA; l Department of Pathology and Laboratory Medicine, Perelman School of Medicine, University of Pennsylvania, Philadelphia, PA, USA

**Keywords:** Brain Extraction, Skull-stripping, Brain tumor, Glioma, Glioblastoma, Deep learning, Evaluation, TCIA

## Abstract

Brain extraction, or skull-stripping, is an essential pre-processing step in neuro-imaging that has a direct impact on the quality of all subsequent processing and analyses steps. It is also a key requirement in multi-institutional collaborations to comply with privacy-preserving regulations. Existing automated methods, including Deep Learning (DL) based methods that have obtained state-of-the-art results in recent years, have primarily targeted brain extraction without considering pathologically-affected brains. Accordingly, they perform sub-optimally when applied on magnetic resonance imaging (MRI) brain scans with apparent pathologies such as brain tumors. Furthermore, existing methods focus on using only T1-weighted MRI scans, even though multi-parametric MRI (mpMRI) scans are routinely acquired for patients with suspected brain tumors. In this study, we present a comprehensive performance evaluation of recent deep learning architectures for brain extraction, training models on mpMRI scans of pathologically-affected brains, with a particular focus on seeking a practically-applicable, low computational footprint approach, generalizable across multiple institutions, further facilitating collaborations. We identified a large retrospective multi-institutional dataset of *n* = 3340 mpMRI brain tumor scans, with manually-inspected and approved gold-standard segmentations, acquired during standard clinical practice under varying acquisition protocols, both from private institutional data and public (TCIA) collections. To facilitate optimal utilization of rich mpMRI data, we further introduce and evaluate a novel “modality-agnostic training” technique that can be applied using any available modality, without need for model retraining. Our results indicate that the modality-agnostic approach^1^ obtains accurate results, providing a generic and practical tool for brain extraction on scans with brain tumors.

## 1. Introduction

Brain extraction, also known as skull-stripping, describes the process of removing the skull and non-brain tissues (e.g., neck fat) from brain magnetic resonance imaging (MRI) scans. It is a crucial step for pre-processing neuro-imaging datasets and has an immediate bearing on all subsequent investigative procedures. It is also a necessary processing step in most studies for compliance with privacy-preserving regulations, such as the Health Insurance Portability and Accountability Act of 1996 (HIPPA) and the General Data Protection Regulation of 2016 (GDPR). The effects of brain extraction on various analyses have been reported in the literature, including brain tumor segmentation (Menze et al., 2015; Bakas et al., 2018), lesion segmentation (Winzeck et al., 2018), cerebral hemisphere segmentation (Zhao et al., 2010), methods for surgical interventions (Leote et al., 2018; Nadkarni et al., 2015), neuro-degeneration (Gitler et al., 2017), devising radiation therapy (Kim et al., 2019), image registration (Woods et al., 1993; Klein et al., 2010), predicting Alzheimer’s disease (Frisoni et al., 2010), multiple sclerosis (Radue et al., 2015), estimation of cortical thickness (Haidar and Soul, 2006; MacDonald et al., 2000), and cortical surface reconstruction (Dale et al., 1999; Tosun et al., 2006).

Manual delineation of the brain (Souza et al., 2018) is very laborious and time-consuming, resulting in inter- and intra-rater variations and affecting reproducibility in future applications. Multiple automated methods have been developed over the years to overcome these shortcomings (Smith, 2002; Segonne et al., 2004; Eskildsen et al., 2012; Doshi et al., 2013; Shattuck et al., 2001; Iglesias et al., 2011). In recent years, DL methods, and particularly Convolutional Neural Networks (CNNs), have obtained state-of-the-art results in multiple problems of image segmentation.

**Fig. 1.**
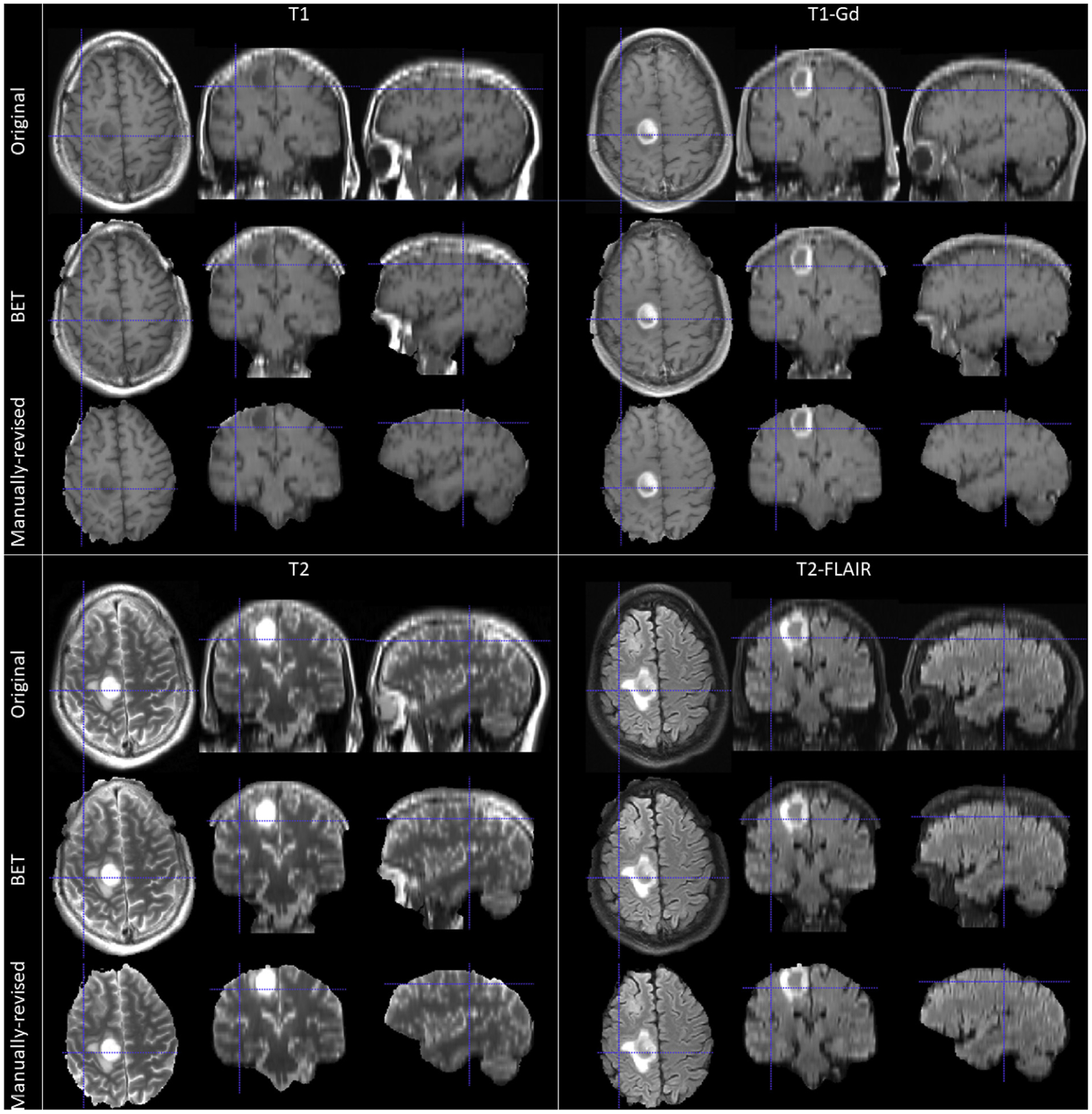
Example 2D tri-planar sections of mpMRI brain tumor scans after using the brain extraction tool (BET) (Smith, 2002) and followed by manual revisions.

Importantly, most existing computational methods for brain extraction were developed for and evaluated on brain scans without apparent pathologically-affected regions such as tumors or lesions, and they typically use only the T1-weighted MRI scans. Scans with brain tumors may present an important challenge for supervised machine learning algorithms that exclusively train on scans of healthy subjects, causing them to fail, particularly in the proximity of the tumor regions. Suboptimal brain extraction on these scans may result in parts of the skull and neck, which usually have tumor-like radiographic characteristics, being included on the final brain mask, or tumor regions being considered as non-brain, hence in both cases adversely affecting consecutive analyses. Example snapshots of such problematic brain extraction using the brain extraction tool (BET) (Smith, 2002) are shown in Fig. 1.

Another important challenge of brain extraction on multi-institutional, multi-parametric MRI (mpMRI) scans with tumors is the complexity and variability of the imaging data. Brain tumor scans acquired during standard clinical practice or even research studies and clinical trials, include a lot of sources of variation affecting their intrinsic radiographic characteristics/appearance, which may not be perceivable by visual observation but even the sub-visual differences affect further computational analyses. Examples of sources of variation include, but are not limited to imaging modalities, quality of the scans, scan acquisition parameters, e.g., repetition time (TR), echo time (TE), flip angle, slice thickness. Hence, robustness of algorithms to imaging variations and their generalizability to available imaging modalities are important requirements for achieving widespread application in clinical and research settings.

Finally, a third important requirement for the field is to have tools that are easy to deploy and apply, and with a low computational footprint. In methodological research studies, obtaining the highest performance is often the main goal, undermining other concerns such as complexity, running time and portability of a new method. However, if the goal is clinical translation and widespread usage, methods should be designed for being used by a variety of users with different hardware and software requirements, as well as competency levels.

In this study, we sought to provide a tool that addresses the specific challenges of brain extraction on scans with brain tumors, with a particular focus on coming up with a practical approach that is applicable and generalizable across multiple institutions and multiple modalities, further facilitating collaborations. Towards this aim, we performed a comprehensive performance evaluation of recently established DL architectures for semantic segmentation. We evaluated 5 widely used DL network architectures, using multiple datasets and various cross-validation strategies. Evaluation data included mpMRI brain tumor scans acquired during standard clinical practice under varying acquisition protocols from 3 private institutions, as well as publicly available data from The Cancer Imaging Archive (TCIA) (Clark et al., 2013; Scarpace et al., 2016; Pedano et al., 2016; Bakas et al., 2017a, 2017b, 2017c), with corresponding gold standard brain masks for all scans. DL architectures were trained using different combinations of input image modalities, including training on each single modality individually, multi-modality training and ensembles of models trained on single modalities. Importantly, we introduced a novel “modality-agnostic training” technique to enable brain extraction on new scans using any available modality, without the need to retrain the model. The proposed modality-agnostic training, which brings noticeable advantages for widespread application, obtained promising results in our comparative evaluations against models trained using other top-performing input modality combinations.

## 2. Materials and methods

### 2.1. Data

mpMRI scans are routinely acquired across institutions for patients with suspected brain tumors. Scanning protocols, i.e., scanner models, acquired modalities, and scan parameters, vary across institutions, as well as depending on the time of scan with respect to patient’s treatment history. In this study, we included four structural modalities that are commonly acquired at baseline pre-operative time-point: native (T1) and post-contrast T1-weighted (T1Gd), native T2-weighted (T2), and T2-weighted Fluid Attenuated Inversion Recovery (Flair) MRI scans.

We identified 3; 220 retrospective multi-institutional mpMRI scans from both private and public collections. The private retrospective collections included 2; 520 MRI scans from the hospitals of a) the University of Pennsylvania (UPenn, *n* = 1; 812), b) Thomas Jefferson University (TJU, *n* = 608), c) MD Anderson Cancer Center (MDA, *n* = 100). The public data are available through TCIA (Clark et al., 2013) and comprise of the scan collections of a) The Cancer Genome Atlas Glioblastoma (TCGA-GBM, *n* = 328) (Scarpace et al., 2016) and b) The Cancer Genome Atlas Lower Grade Glioma (TCGA-LGG, n = 372) (Pedano et al., 2016), with their corresponding brain masks as provided through the International Brain Tumor Segmentation (BraTS) challenge (Menze et al., 2015; Bakas et al., 2017a, 2017b, 2017c, 2018). Notably, the bra in masks used in the BraTS challenge were prepared primarily focusing on the tumor area, rather than the overall brain. Accordingly, we performed an additional manual inspection on the BraTS dataset and we excluded scans with brain masks that were missing parts of the brain. The final dataset included 3; 220 mpMRI scans from 805 subjects, with the 4 structural modalities previously mentioned for each subject (Table 1). The ground truth brain masks of all the included scans were obtained through semi-automatic annotation using the ITK-SNAP (itksnap.org) software (Yushkevich et al., 2006; Yushkevich et al., 2019) and approved by a board-certified neuro-radiologist (M.B.) with more than 10 years of experience working with gliomas.

Importantly, this multi-institutional data collection was highly heterogeneous with a wide range of variability in scan quality, slice thickness of different modalities, scanning parameters, as well as pre-processing of initial scans, e.g (Table 1). Briefly, the UPenn data included T1 scans with high axial resolution. MDA data had similar characteristics, and accordingly it was used to evaluate the generalizability of other similar institutional data. All scans in TJU data had lower resolution compared to other datasets.

**Fig. 2.**
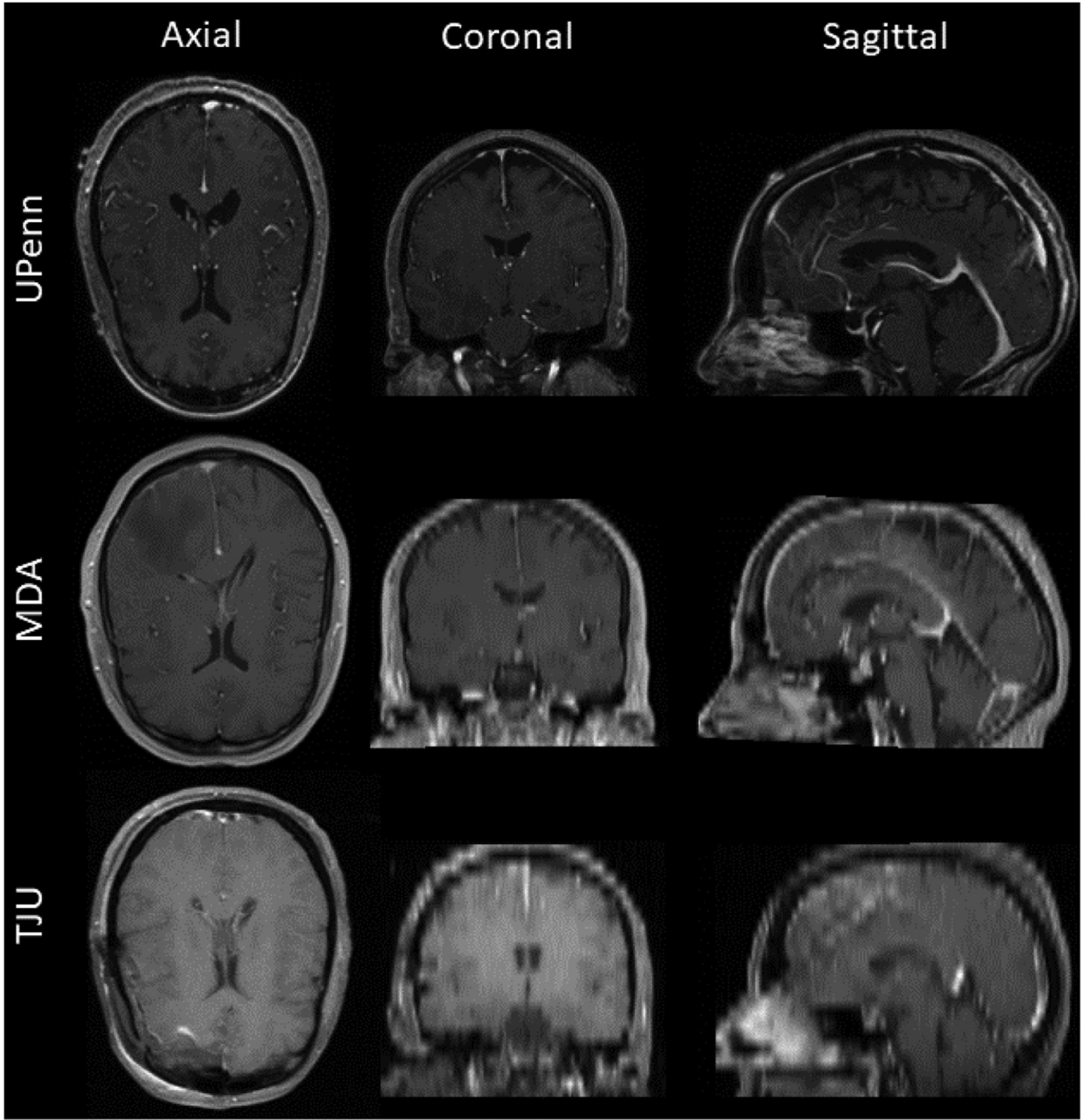
Example 2D tri-planar sections of T1Gd MRI scans from UPenn, MDA, and TJU datasets, respectively in each row, illustrating the high variability between datasets. Note the lower resolution of the TJU scans emphasizing the resampling interpolation.

Examples of scans for each institution are shown in Fig. 2, showcasing the specific challenges of each dataset.

### 2.2. Pre-processing

**Fig. 3.**
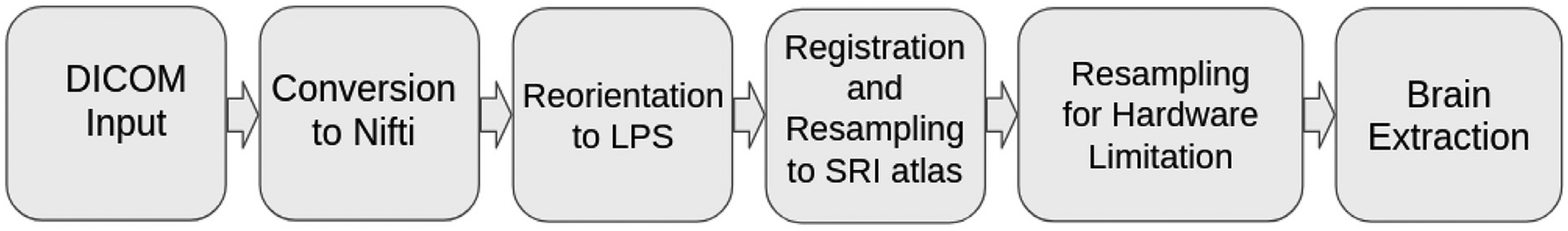
Overview of the complete framework applied in this study leading to results for further analyses.

Since the scans included in this study were heterogeneously obtained from different scanners and acquisition protocols, they all underwent the same pre-processing protocol to make image dimensions and voxel sizes uniform across studies and modalities. Specifically, all DICOM scans were converted to the NIfTI (Cox et al., 2004) file format and then, following the well-accepted^2^ pre-processing protocol of the BraTS challenge (Menze et al., 2015; Bakas et al., 2017a, 2017b, 2017c; Bakas et al., 2018), the T1Gd scan of each patient was rigidly registered and resampled to an isotropic resolution of 1 *mm*^3^ based on a common anatomical atlas, namely SRI (Rohlfing et al., 2010). The remaining scans (i.e., T1, T2, Flair) of each patient were then rigidly co-registered to this resampled T1Gd scan. The *Greedy* registration algorithm (Yushkevich et al., 2016) was used for all registrations. In order to accommodate hardware limitations two additional steps were considered. First, all scans were zero-padded with 3 and 2 slices on the top and bottom of the axial direction, ensuring that the image size on each dimension is factorized by 2, i.e., all scans converted from the SRI image size of 240×240×155 to 240 × 240 × 160 voxels. Subsequently, all scans were down-sampled to an image size of 128 × 128 × 128, converting their SRI isotropic voxel resolution (1:0 × 1:0 × 1:0*mm*^3^) to anisotropic (1:875× 1:875× 1:25*mm*^3^). These scans were finally used for training all the DL architectures. An overview of the pre-processing applied in this study is shown in Fig. 3.

### 2.3. DL network architectures

Multiple DL network architectures were tested in our comparative performance evaluation. Our selection was mainly motivated by their wide application on other segmentation tasks. The specific architectures included here comprise a) 2D U-Net (Ronneberger et al., 2015; Doshi et al., 2019), b) 3D U-Net (Çiçek et al., 1606; Milletari et al., 1606), c) Fully Convolutional Network (FCN) (Long et al., 1411), d) DeepMedic (Kamnitsas et al., 1603), and e) 3D U-Net with Residual connections (3D-ResU-Net) (Drozdzal et al., 1608; He et al., 1512). Below, we provide a very brief overview of each architecture, with references for more detailed descriptions, and we describe the specific parameters and network design that was used in our experimental evaluations. Notably, to address hardware limitations on memory utilization and keep the requirements to < 12GB, we utilized Instance Normalization (Ulyanov et al., 1607) instead of Batch Normalization (Ioffe and Szegedy, 1502) for training the 3D network architectures.

#### 2.3.1. 2D U-Net

U-Net (Ronneberger et al., 2015) was originally proposed in 2015 and its numerous variations have been used successfully in various segmentation tasks delivering promising results. In this study we include a heavily involved variation of a 2D U-Net (Doshi et al., 2019), including residual connections and an inception module. We refer to this architecture as “2D-Res-Inc”. Specifically, the network architecture consists of an encoding path and a corresponding decoding path, as in U-Net (Ronneberger et al., 2015), followed by a voxel-wise, multi-class soft-max classifier to produce class probabilities for each voxel independently. A major modification in this implementation is the use of branched convolutions, adapted from the Inception-ResNet-v2 architecture (Szegedy et al., 2017). This module ensures that each branch learns a different representation of the input features by learning both shallow and deeper features, and allows the subsequent layer to abstract features from different scales simultaneously. This property can be extremely useful when dealing with segmentation tasks for more complex or heterogeneous structures.

#### 2.3.2. 3D U-Net

The 2D U-Net was extended in 3D volumetric variations in 2016 (Çiçek et al., 1606; Milletari et al., 1606). Here we utilize a 3D U-Net (Çiçek et al., 1606) with a customized image input size of 128 128 × 128 × 128. Furthermore, to accommodate hardware limitations, we use 16 base filters, compared to the original implementation that included 64. The pattern for a single convolution block was given as 3D Convolution operation (3 × 3 × 3) followed by Leaky ReLU (LReLU) and Instance Normalization with running statistics during learning. Adam optimizer was used with a learning rate of 0.01 over 25 epochs. The number of epochs was determined according to the amount of improvement observed. Notably, we evaluated the basic 3D-U-Net architecture in terms of the initial numbers of filters and their relation to the segmentation quality. Details of this analysis, showing the trade off between hardware requirements and performance, can be found in the supplementary section.

#### 2.3.3. 3D U-Net with residual connections

**Fig. 4.**
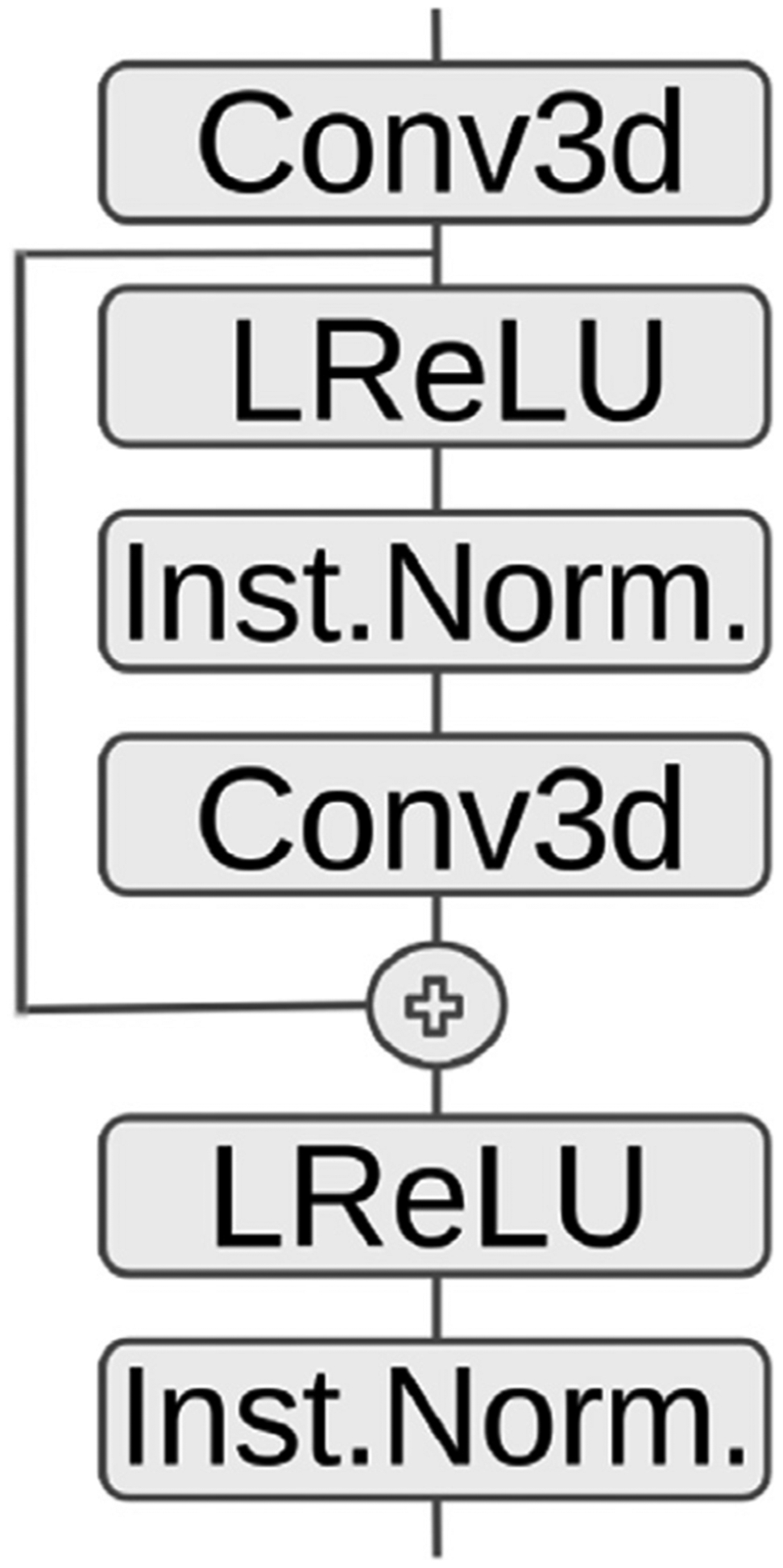
Residual Connection in Encoder/Decoder block for our 3D-Res-U-Net implementation.

We extended 3D U-Net, by applying residual connections improving the backpropagation process (Drozdzal et al., 1608; He et al., 1512). Our 3D-Res-U-Net implementation follows the principle of the 3D U-Net architecture, while further including skip connections in every convolution block, differently from other methods that use a 3D UNet. A schematic that illustrates these operations is shown in Fig. 4. Similar to 3D-U-Net above, we used Adam optimizer with a learning rate of 0.01 over 25 epochs, and the number of epochs was determined according to the amount of improvement observed.

#### 2.3.4. FCN

The FCN architecture (Long et al., 1411), which was introduced in 2017, captures hierarchical features, imaging patterns, and spatial information of each input image with receptive fields. FCN is considered less computationally expensive than other architectures, due to not having an “expensive” decoding part as U-Net, and hence provides faster coarse segmentation of various problems (Litjens et al., 2017). Here, we apply a 3D FCN. Similarly to other architectures, the Adam optimizer was used with a learning rate of 0.01 over 25 epochs. Number of epochs was determined according to the amount of observed improvement.

#### 2.3.5. Deep-medic

DeepMedic (Kamnitsas et al., 1603) was originally proposed in 2015, as the first 3D architecture, when it emerged as a winner of the ISchemic LEsion Segmentation (ISLES) challenge (Winzeck et al., 2018). The standard DeepMedic architecture, as provided in its GitHub repository^3^ is a 3D CNN with a depth of 11-layers, and a double pathway to provide sufficient context and detail in resolution. In our evaluation, we applied the original version of DeepMedic^4^ with the default parameters provided, and we applied a hole-filling algorithm as a post-processing step.

### 2.4. Experimental design

Considering the complexity of the task, i.e., the combinatorial explosion of experiments using multiple sites, train/test combinations, input modalities and network architectures, we followed a multi-step experimental design, focusing on each step on a specific objective, as described below.

All hyper-parameters stayed consistent throughout all the experiments. Each of the applied architectures needed different time for convergence, based on their individual parameters. Table 2 shows the time each architecture run until convergence during training on a compute node equipped with an NVidia P100 GPU, with 12 GB memory.

#### 2.4.1. Comparative evaluation of DL network architectures and input data combinations

On this first step of our evaluations, we performed an exhaustive comparison of various network architectures and input image combinations. Specifically, models of all architectures were trained solely on data from a single institution (i.e., UPenn) and then inferred on datasets of multiple institutions (i.e., UPenn, TJU, and MDA). During the training phase, a hold-out cohort of 180 mpMRI scans from 45 UPenn subjects were used for internal algorithmic validation and convergence evaluation. Each method was trained using as input either single individual scans or combined mpMRI scans, as described in Section 2.5. A total of 7 input image combinations configurations were evaluated: a) four combinations of individual modalities (i.e., T1-T1, T1Gd-T1Gd, T2-T2, Flair-Flair), b) ensemble of these individual modalities based on majority voting, and c) two mpMRI combination, including the combination of T1 and T2 (Multi-2), as well as the combination of all four individual modalities (Multi-4).

In our attempt to get a more complete picture, we have also compared these DL architectures with BET and FreeSurfer, which can be considered two of the most widely used traditional approaches for brain extraction. For this comparison we only utilized the T1 scans that BET and FreeSurfer can utilize. Furthermore, to showcase how the disease can affects the performance of DL algorithms we have trained a DL model on scans from 50 healthy subjects (2D-Res-Inc-H) acquired for different studies and applied it in the same brain tumor scans we used for this step of the evaluation. The ground truth labels of the healthy brain scans were generated from different raters and confirmed for obvious errors by an expert data scientist with experience working in neuroimage analysis for more than 9 years.

#### 2.4.2. Modality-agnostic training

On the second step, we evaluated the performance of a “modality-agnostic training technique”, which we developed aiming to provide a tool that is widely applicable to datasets with different MRI modality combinations. A similar concept was also recently presented by (Isensee et al., 2019), which shows the relevance of the proposed work. The modality-agnostic models were constructed by using different modalities as independent input samples during the training, such that a single model learns the mapping between any single image modality type and the target brain mask segmentation, without necessarily being informed about the type of modality provided as input. The modality-agnostic training was implemented and validated using architectures selected based on the performance evaluation of step 1.

#### 2.4.3. Performance effect of training on diverse data

The size and diversity of the training dataset are major factors affecting the performance and generalizability of DL models. In order to evaluate the effect of multi-site training on segmentation accuracy we performed additional experiments using models trained exclusively on data from a single institution (UPenn only) against models trained on multi-site data, i.e., UPenn, TJU, and MDA. For both cases, we used the large multi-institutional TCIA dataset for testing, thus applying the final models on a highly heterogeneous and previously unseen dataset, which allowed us to evaluate the generalizability of the proposed DL models.

### 2.5. Input data combination strategies for training

#### 2.5.1. Training on individual modalities

Following the current literature on brain extraction methodologies, where T1 is solely used, we trained every architecture on T1. Additionally we trained models on each other available individual MRI modality, to compare the potential contribution of each modality beyond T1. In the remainder of this manuscript we refer to these models using the notation “modality-modality” to indicate that a single modality was used for both training and inference. For example we refer to a 3D U-Net trained and inferred on T1, as T1-T1 3D-U-Net.

We should note that the performance of models trained on individual modalities was also evaluated in ensemble configurations.

#### 2.5.2. Training on multiple mpMRI modalities

To take advantage of the richness of the routine mpMRI acquisitions for brain tumor patients, we also considered training methods on multiple modalities, instead of training on single modality individually. We limited these experiments to two main configurations: a) combining T1 and T2, that we refer to as “Multi-2”, and b) combining all 4 structural mpMRI modalities together that refer to as “Multi-4”. The first configuration (Multi-2) is selected as it was previously shown that T2 contributes to improved skull-stripping performance (Bakas et al., 2017a) and can be applied on cases where gadolinium is not considered, i.e., in non-tumor cases.

#### 2.5.3. Modality-agnostic training

We propose a novel approach that provides a “hybrid” alternative to single-modality and multi-modality training techniques, aiming to optimally use multi-modal imaging data, while not necessarily requiring existence of a pre-defined set of modalities. Specifically, in this configuration, a DL model is trained using all available modalities for a subject. However, in contrast to the multi-modality approach where the combination of all modalities from a subject is used as a single data sample, we feed each modality to the network as an independent data sample. This process attempts to contribute on forcing the network to learn the brain shape prior, instead of texture priors that CNNs usually learn (Geirhos et al., 1811). In this way, the model allows making an inference from a single modality, while being trained on multi-modal data. Importantly, during the inference, the model does not need to know which modality was provided as the input scan. The most important practical benefit of this approach is its robustness to missing data, i.e., it can be applied directly even if a specific modality is missing or is not usable due to low scan quality.

### 2.6. Evaluation metrics

Following the literature on semantic segmentation we use the following metrics to quantitatively evaluate the performance of the trained methods. We have further utilized the metrics of sensitivity and specificity in the supplementary material.

#### 2.6.1. Sørensen-dice similarity coefficient

The Sørensen-Dice Similarity Coefficient (*Dice*) is commonly used to evaluate and report on the performance of semantic segmentation. *Dice* measures the extent of spatial overlap, while taking into account the intersection between the predicted masks (*PM*) and the provided ground truth (*GT*), hence handles over- and under-segmentation. *Dice* can be mathematically defined as:

(1) $$\text{Dice}=\frac{2\left|GT\cap PM\right|}{\left|GT\right|+\left|PM\right|}*100$$

where it would range between 0 and 100, with 0 describing no overlap and 100 perfect agreement.

#### 2.6.2. Hausdorff

While the *Dice* score is the most commonly used metric for comparing two segmentation masks, it is not sensitive to local differences, as it represents a global measure of overlap. For the specific problem of brain extraction, local differences may be very important for properly assessing the quality of the segmentation. Accordingly, we calculated a complementary metric, the *Hausdorf f* distance, which measures the maximum distance of a point set to the nearest point in another (Rockafellar and Wets, 2005):

(2) $$h(A,B)={\text{max}}_{a\in A}{\text{min}}_{b\in B}\|a-b\|$$

We specifically calculated the 95th percentile of the *Hausdorf f* distance between the contours of the two segmentation masks, a robust measure of the maximum distance between the segmentations.

## 3. Results

### 3.1. Comparative evaluation of network architectures with BET and FreeSurfer using T1 image as input

**Fig. 5.**
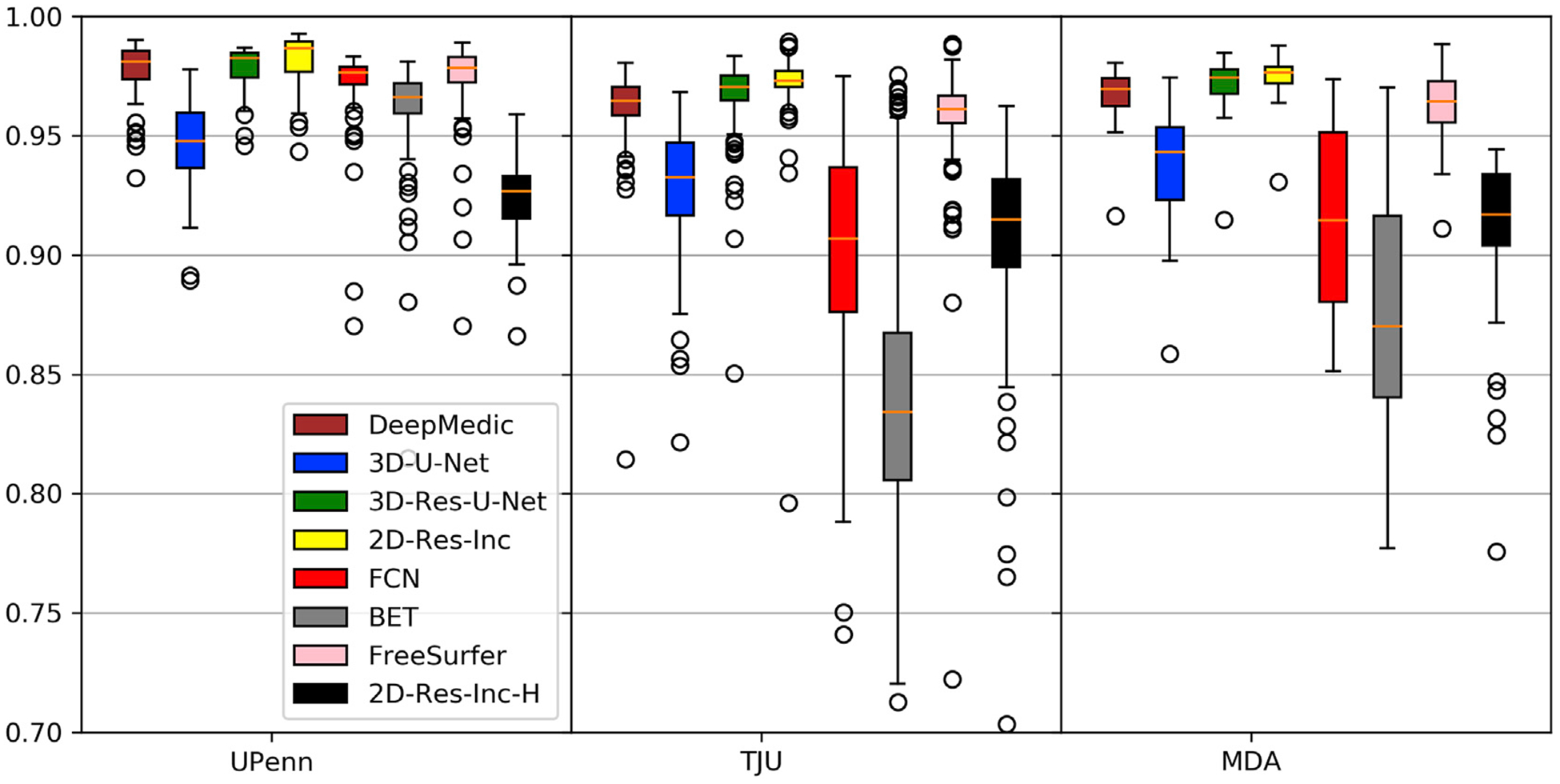
Quantitative evaluation of various DL network architectures compared with BET and FreeSurfer, using the T1 MRI brain tumor scans.

Considering the general approach in the field, we first compared different architectures, as well as BET (Smith, 2002) and FreeSurfer (Segonne et al., 2004), two of the most widely used brain extraction tools, using only the T1 scan for brain extraction (T1-T1 models). Dice scores obtained by different methods on 3 datasets are shown in Fig. 5. The DL-based algorithms (regardless of the architecture) significantly outperformed BET, with a single exception for the UPenn dataset. We also note that FreeSurfer is superior to BET, FCN, and 3D-UNet, but is still outperformed by the other architectures.

Furthermore, based on the results shown in Fig. 5, we can confirm with certainty that the presence of diffuse gliomas in MRI scans indeed affect the performance of DL models trained on healthy brain scans. Specifically, we note that the best performing DL model for brain extraction in T1 brain tumor scans (2D-Res-Inc - Fig. 5) ends up with the lowest performance among the DL architectures when trained on healthy brain T1 scans (2D-Res-Inc-H - Fig. 5), with its *Dice* showing a decrease of at least 6% across the data from the multiple institutions.

### 3.2. Comparative evaluation of network architectures using different input data combinations

**Fig. 6.**
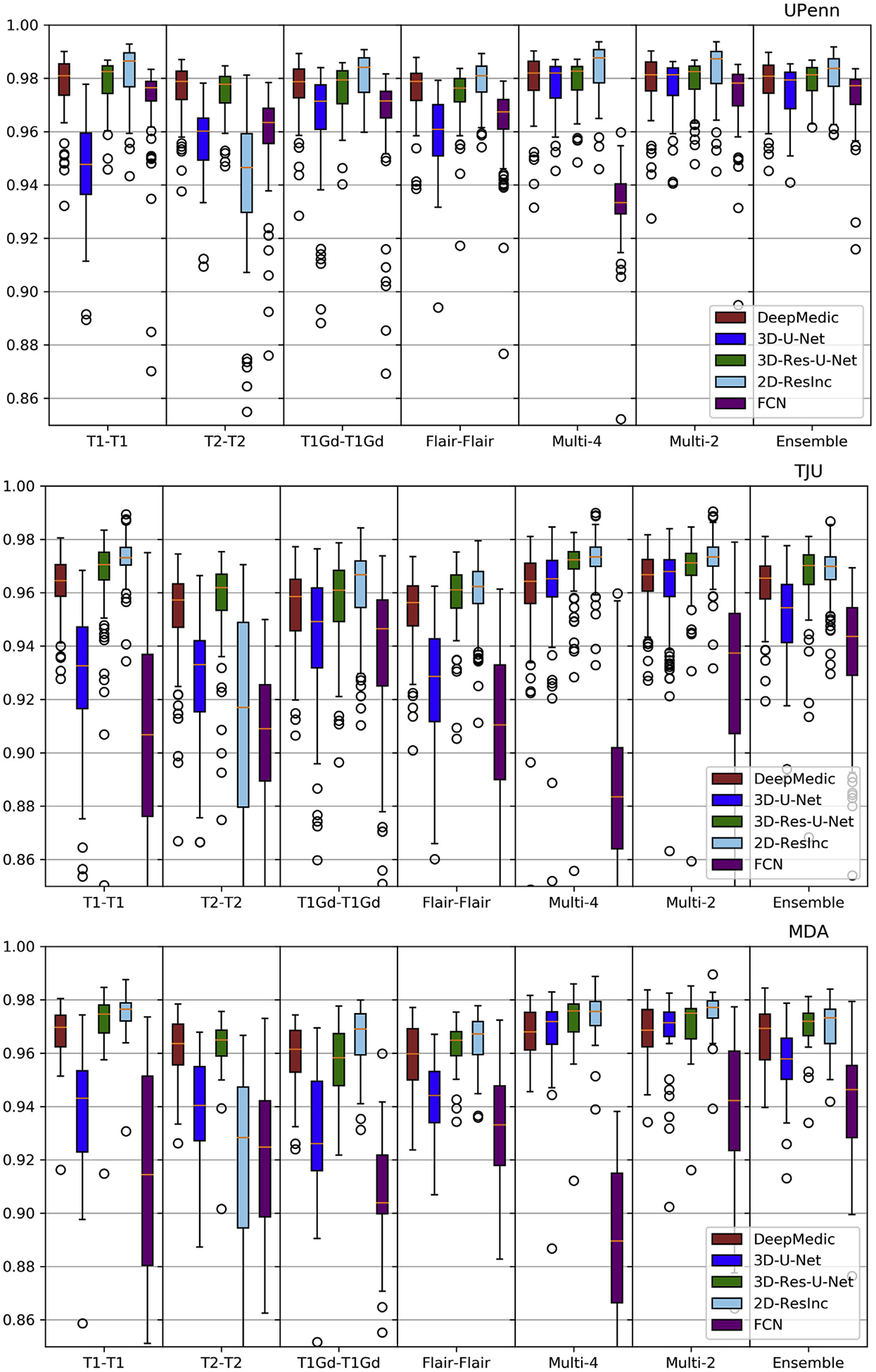
Quantitative (average *Dice*) evaluation of various DL network architectures. From top to bottom rows we see results on the data from (*a*) UPenn, (*b*) TJU, and (*c*) MDA. The evaluated models in this figure include training on individual modalities and their ensemble using majority voting, as well as multi-modality training.

**Fig. 7.**
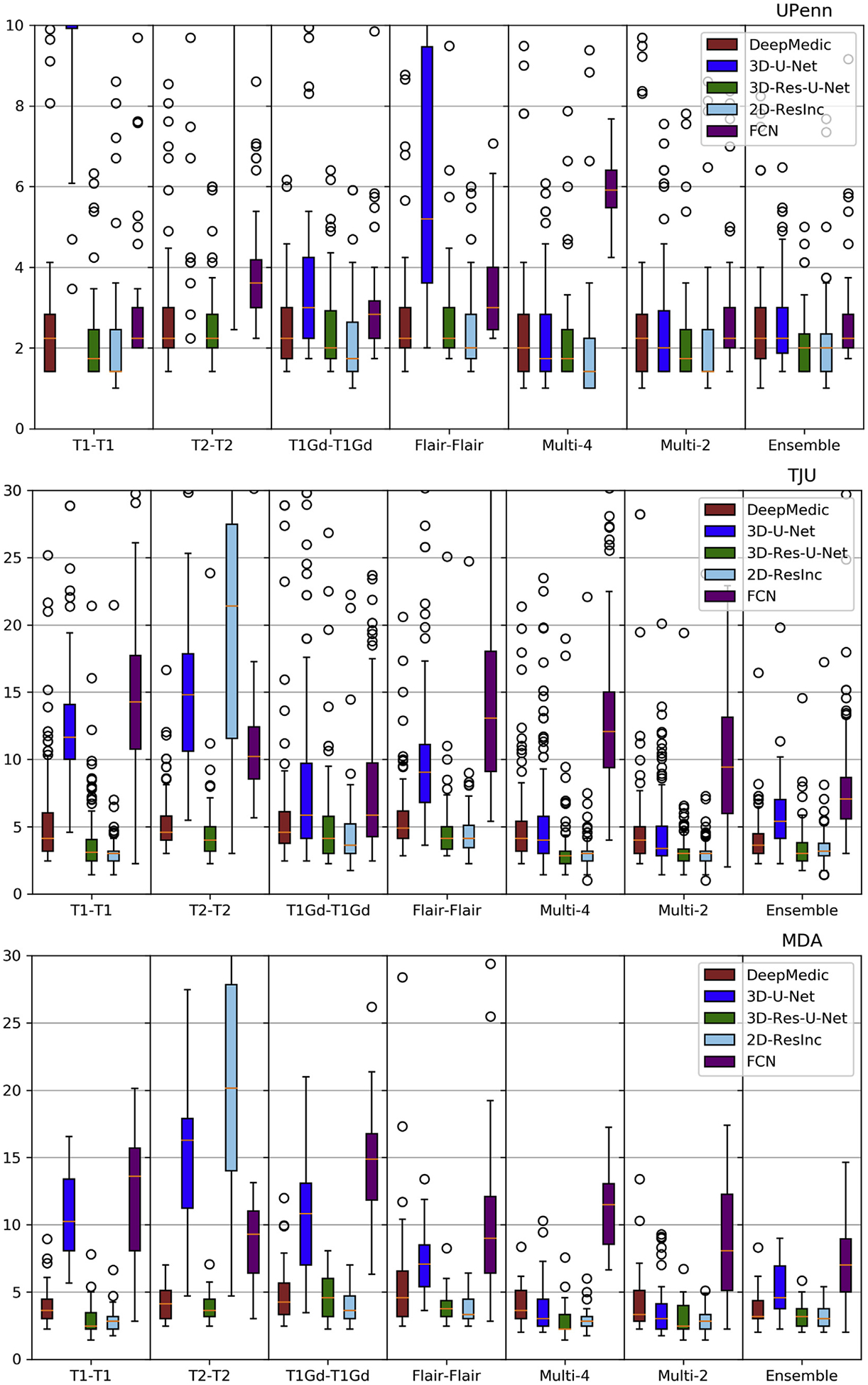
Quantitative (average *Hausdorf f*) evaluation of various DL network architectures. From top to bottom rows we see results on the data from (*a*) UPenn, (*b*) TJU, and (*c*) MDA. The evaluated models in this figure include training on individual modalities and their ensemble using majority voting, as well as multi-modality training.

Plots of average *Dice* and *Hausdorf f* metrics for scans from the 3 different institutions obtained using each model are shown in Figs. 6–7 and S. Tables 3–4 (average Sensitivity and Specificity metrics are shown in Supplementary Figs. 13 and 14).

We observed that the 2D-ResInc model was consistently outperforming other models, although marginally, on all datasets, with the exception of the “T2-T2” and the “Ensemble model”. DeepMedic and 3D-Res-U-Net models obtained comparable, but slightly lower, performance. On the other hand, 3D-U-Net, and FCN showed lower performance, with FCN obtaining the lowest accuracy for most input configurations.

From all input data configurations, “T1-T1” and “Multi-4” models obtained higher performance, with consistent results across datasets and network architectures, although there are few exceptions. Single modality training using modalities other than T1 resulted in lower performance, while the “Ensemble” models did not improve the results beyond the performance of the “T1-T1” model. “Multi-4” training showed improved performance, obtaining the best results comparable to the “T1-T1” model.

We have further conducted a statistical analysis using Wilcoxon tests, where we compared the *Dice* of the 3D-Res-U-Net with every other architecture, across all datasets and input data combinations. As previously mentioned and indicated in Figs. 6–7 and S.Tables 3–4, 3D-ResU-Net has consistently the smallest differences in *Dice* when compared to Deep-Medic, across all models. Nevertheless, 3D-ResU-Net is significantly (*p* < 0:041) better than DeepMedic in all datasets and models with the exceptions of i) the cases where 3D-ResU-Net improvements are not significant (i.e., “T2-T2” on MDA data, and “T1-T1”, “T1Gd-T1Gd”, “Multi-2”, and “Ensemble” on UPenn data), ii) “T1Gd-T1Gd” on MDA data where DeepMedic is insignificantly better, and iii) “T2-T2” and “Flair-Flair” models where DeepMedic shows significantly (*p* < 0:0035) improved performance. When comparing 3D-ResU-Net with 2D-ResInc, we note that 3D-ResU-Net is comparable but significantly (*p* < 0:033) worse than 2D-ResInc for all datasets for all models except, i) “T2-T2” where 2D-ResInc fails dramatically and 3D-ResU-Net is significantly (*p* < 1:45*E*^−05^) better, and ii) “Flair-Flair” and “Ensemble” models, where 2D-ResInc is better than 3D-ResU-Net, but with insignificant differences. 3D-ResU-Net is also significantly (*p* < 2:56*E*^−06^) better than FCN for all datasets for all models, and when compared to 3D-UNet, 3D-ResU-Net is significantly (*p* < 0:0025) better for all datasets for all models, except “Multi-4” for the UPenn data, where still 3D-ResU-Net is better than 3D-UNet but with insignificant differences.

Based on the quantitative results of these experiments, we conclude that the 3D-Res-U-Net, DeepMedic, and 2D-ResInc are the three best performing approaches and perform comparably well. The statistical analysis indicated that 2D-ResInc is outperforming 3D-ResU-Net in most cases with the exception of the “T2-T2” model where 2D-ResInc fails dramatically. Furthermore, between 3D-Res-U-Net and DeepMedic we note that 3D-Res-U-Net is better in most models, with consistently the smallest differences in *Dice* between them when compared to the other architectures. Since a major goal of our analysis is also the practicality and footprint of the method both during training and inference, we considered the relatively simpler 3D-Res-U-Net and DeepMedic architectures over the 2D-ResInc for the follow up evaluations.

### 3.3. Modality-agnostic training

Comparative evaluations involved the 3D-Res-U-Net and the Deep-Medic models trained using the modality-agnostic technique, compared against the 3D-Res-U-Net and the DeepMedic using the best performing input combinations in the previous step, i.e., “T1-T1”, “Multi-2”, and “Multi-4” models. Similar to step 1, these models were trained on the same training dataset from UPenn and inference and evaluation was performed using the same three testing datasets. Note that the modality-agnostic model was only trained once using all single modalities sequentially as input. The model was then applied on each modality independently, and the performance was evaluated for each modality separately.

**Fig. 8.**
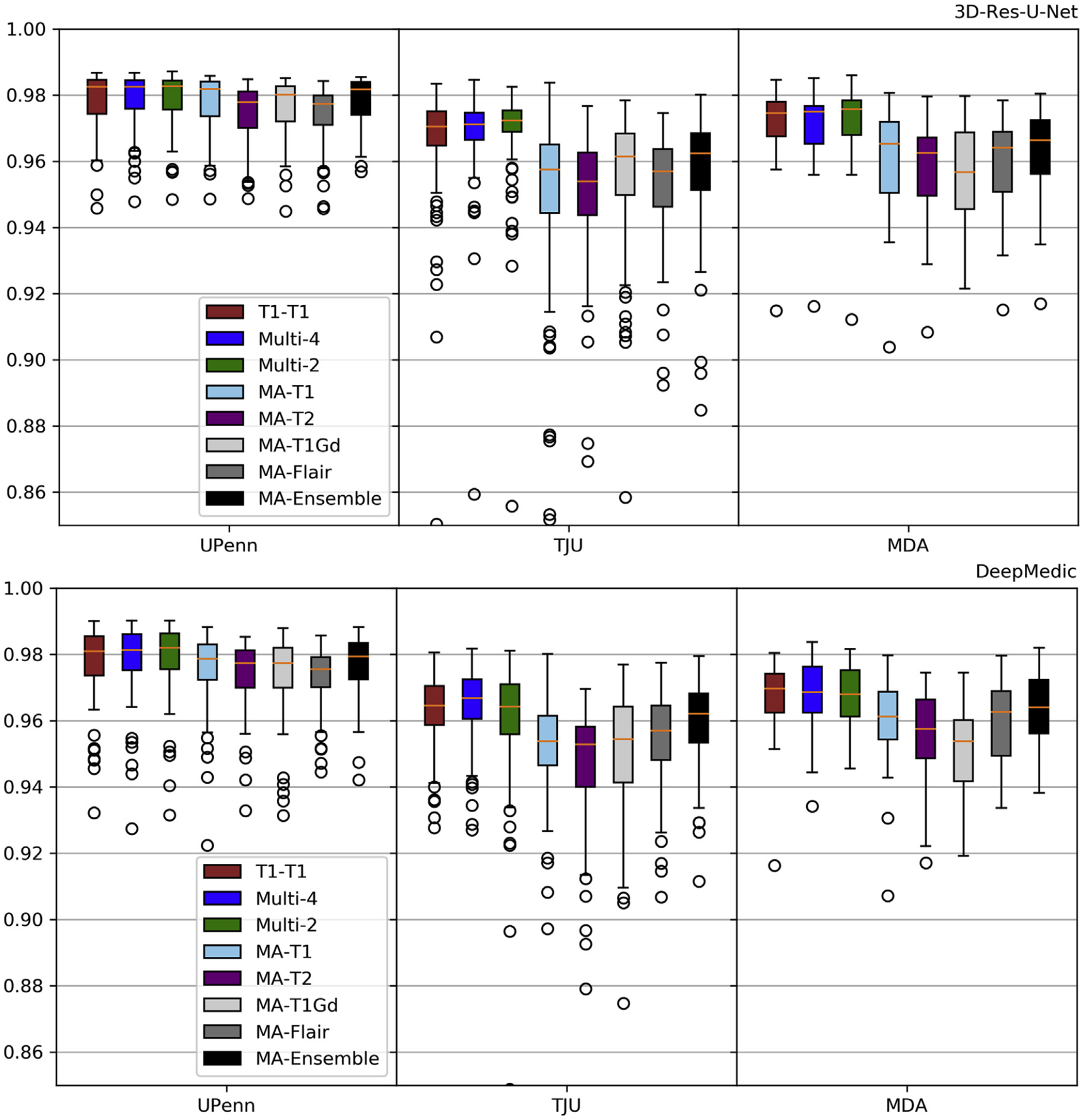
Evaluation results (*Dice*) for the selected 3D-Res-U-Net and DeepMedic on the Modality-Agnostic training process. Results also include training on the best results of Fig. 6 for comparison purposes.

**Fig. 9.**
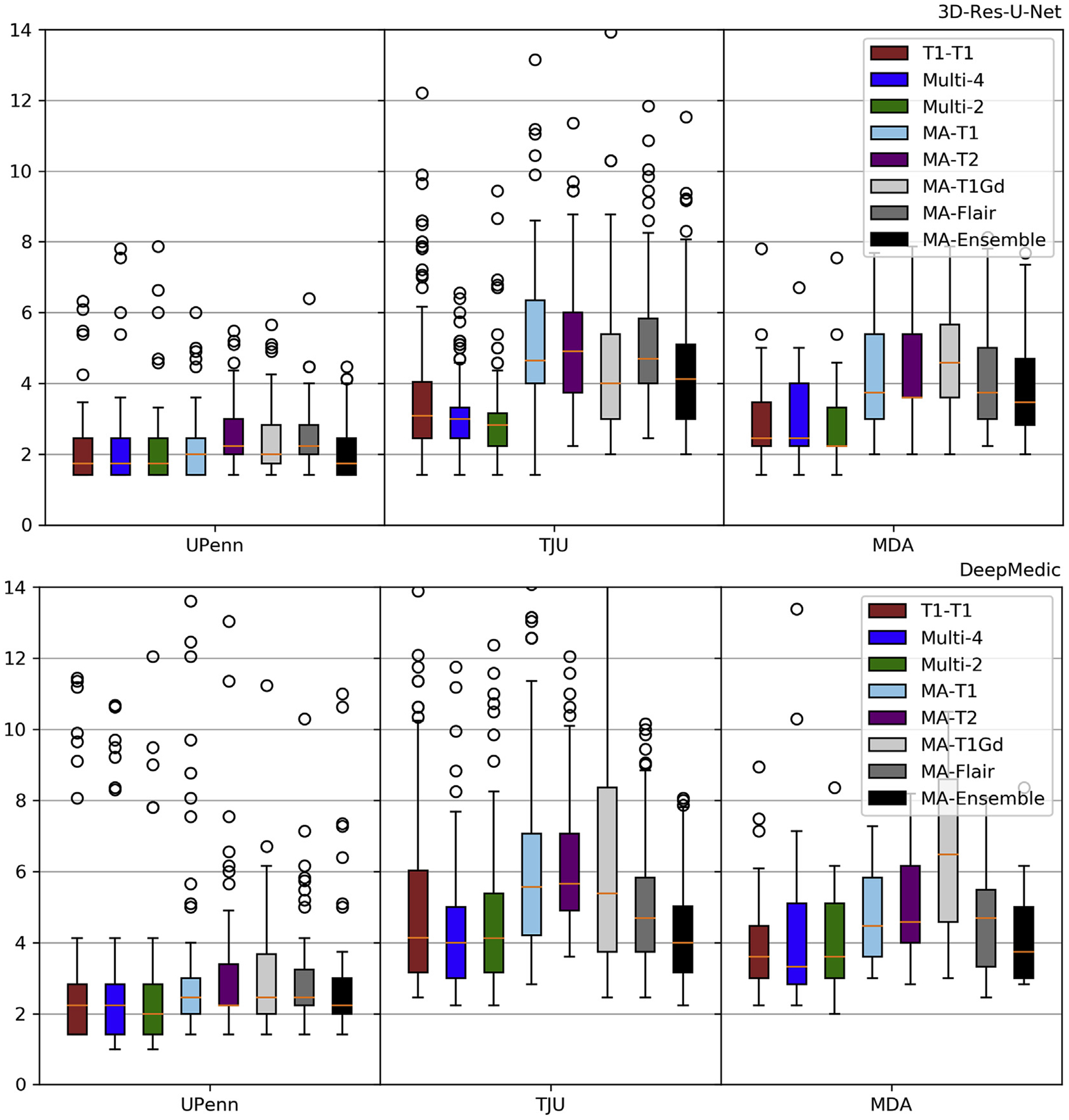
Evaluation results (*Hausdorf f*_95_) for the selected 3D-Res-U-Net and DeepMedic on the Modality-Agnostic training process. Results also include training on the best results of Fig. 7 for comparison purposes.

Comparative results for different models are shown in Figs. 8–9 and S.Tables 3–4, where 3D-Res-U-Net shows a consistent superior performance compared to DeepMedic. The modality-agnostic model performed comparable to “T1-T1” and “Multi-4” models on the UPenn dataset. However, when applied for inference on data from other institutions, i.e., TJU and MDA, we observed gradually inferior performance. Consistently with previous results, the “Ensemble” models did not improve accuracy beyond the performance of the independent modality-agnostic models.

### 3.4. Performance effect of training on diverse data

**Fig. 10.**
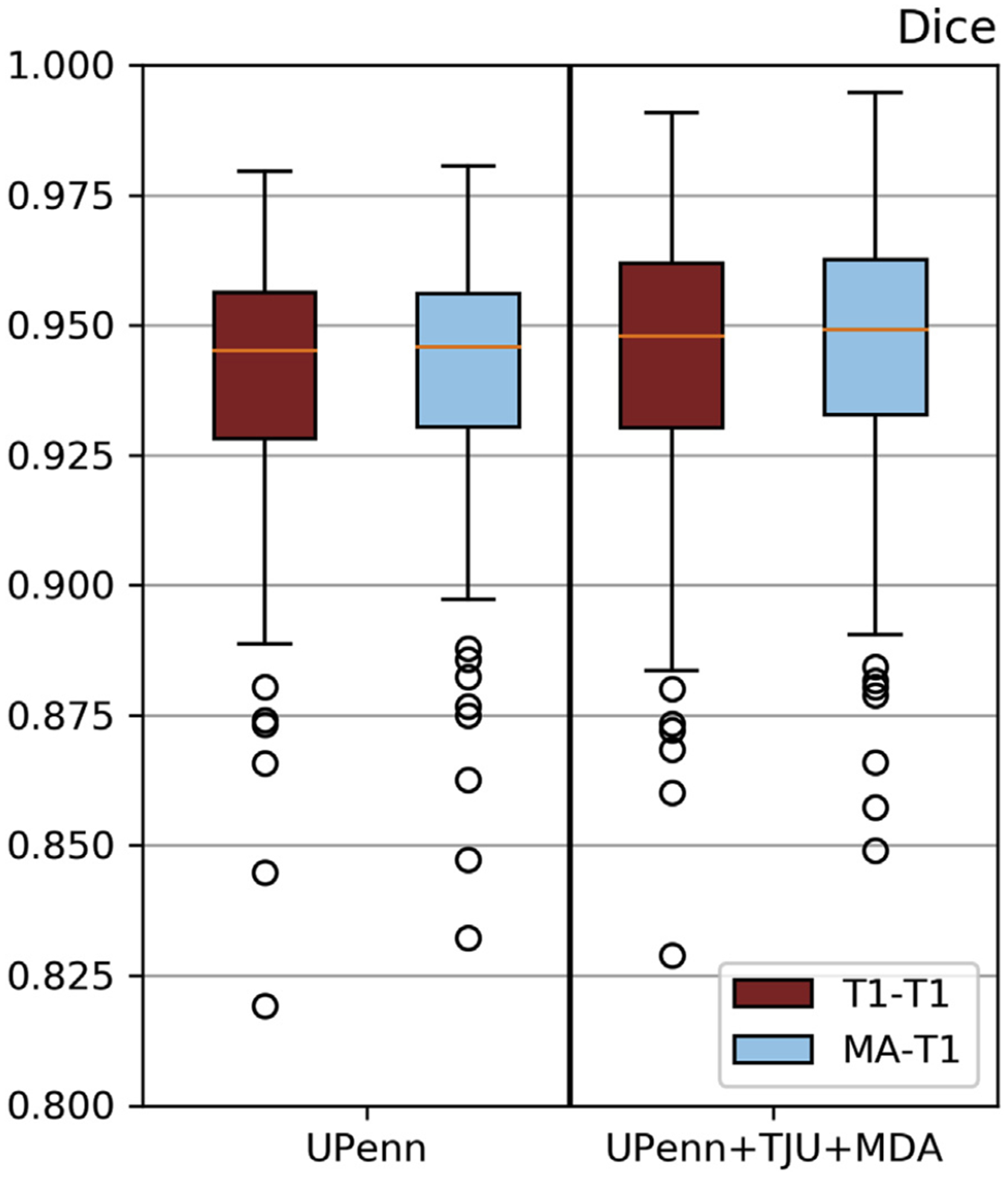
Mean *Dice* of model inference on publicly-available multi-institutional data from TCIA. Diverse data contribute to performance improvements. “M-A” training process performs comparably with “T1-T1”.

We further investigated the effects of training on multi-site data using the combination of UPenn, TJU, and MDA as our training dataset. Trained “T1-T1” and the modality agnostic T1 (“M-A-T1”) models were then evaluated on the independent multi-institutional TCIA dataset (Fig. 10). We observed that training on diverse multi-institutional data (i.e., Fig. 10 - “UPenn + TJU + MDA”) resulted in improved performance, compared to training on data from a single institution (i.e., Fig. 10 - “UPenn”). Importantly, the model trained with the modality agnostic technique performed comparably with the “T1-T1” model using the multi-institutional training data, suggesting the potential of this model as a widely applicable generic tool for segmentation of brain images with tumors.

## 4. Discussion

In this study, we conducted a comprehensive performance evaluation of widely-used DL methods for the task of brain extraction. We selected multiple recent architectures established in the domain of 3D semantic segmentation, while also considering low computational footprint and out-of-the-box implementation availability. We further introduced a novel modality agnostic training technique, preferable to models trained using individual MRI modalities, considering the benefits of its flexibility to availability of different input image modality combinations. The accompanying source code can be found at: https://github.com/CBICA/BrainMaGe.

Many automated methods have previously been developed for brain extraction (Smith, 2002; Segonne et al., 2004; Eskildsen et al., 2012; Doshi et al., 2013; Shattuck et al., 2001; Iglesias et al., 2011). In recent years, several DL-based approaches have also shown promising results in brain extraction (Hwang et al., 2019). Even though these methods are shown to be widely successful on healthy subjects, they tend to be less accurate when evaluated on brain MRI scans with brain tumors. Furthermore, their performance can vary significantly when applied to images from different sites, scanners, and imaging acquisition protocols (Iglesias et al., 2011). Here we evaluated specific DL architectures, considering the availability of their implementation and the corresponding computational footprint. We note that the selected 3D-Res-U--Net and DeepMedic DL methods (as well as others that are not selected but performing similarly, such as the 2D-ResInc) outperformed standard brain extraction methods, such as BET and FreeSurfer. In terms of comparing 3D-ResU-Net, DeepMedic, and 2D-ResInc, we think that the use of branched convolutions with varying depths in the ResInc module makes the 2D-ResInc perform better than the others as it allows the network to simultaneously learn both shallow and deep representations of the region of interest. However, this use of branched convolutions with varying depths makes the 2D-ResInc substantially slower to train and infer when compared to 3D-ResU-Net, as indicated in Table 2. When further comparing the performance of 3D-ResU-Net with DeepMedic, it seems that the residual connections and the larger receptive field (including the whole 3D scan) is what makes the 3D-ResU-Net outperform DeepMedic. Notably, although DeepMedic is the first state of the art 3D CNN, its patch-based approach seems to be the reason for the resulting “holes” in the output predictions (primarily near the ventricles) that make it require a post-processing step as recommended in its original publication (Kamnitsas et al., 1603). This post-processing step is what then renders DeepMedic the second slowest approach following 2D-Res-Inc, with average inference time equal to 17s per scan.

We assessed a total of 7 modality configurations, including both independent MRI scans and combinations of mpMRI brain tumor scans. Amongst these configurations, our results are in line with the existing literature on brain extraction, where the T1-weighted scan is typically used for brain extraction. These results indicate that utilization of the T1 modality (“T1-T1”) achieves the best performance also for scans with brain tumors. However, we should also note that ground-truth labels are generally delineated using the T1 scans, thus creating a potential bias. Ensemble segmentation via majority voting did not contribute in improving performance. The only other model showing comparable performance was the one trained on all 4 modalities, i.e., “Multi-4”. However, between these two alternatives, the “T1-T1” model would be preferred owing to the requirement of only one modality, T1, instead of requiring the co-existence of four modalities to train the “Multi-4” model. Poor and good illustrative examples are shown in supplementary figures 15–16. Poor segmentations are randomly chosen from the data of each institution separately, across all algorithms and after setting a threshold on the *Dice* score as *Thr* < 80%. Similarly, good segmentations were randomly chosen using a threshold on *Dice* score as *Thr* > 98%.

We introduced an alternative novel modality-agnostic brain extraction approach, which is not dependent on any particular modality and may be trained on any available modality. There are at least two reasons why training the algorithm and running it on a limited number of modalities is essential in clinical applications. First, some clinical MRI brain protocols do not include all four modalities. For example, at our institution, the GammaKnife protocol consists only of T2-FLAIR and T1-Gd scans, and hence skull stripping would have to be performed using at most two modalities. The other reason pertains to processing speed during inference, where brain extraction and further analysis has to be performed real time (typically before neuroradiologists open those cases from their worklist). Therefore the time needed for pre- and post-processing is directly affecting the radiological reading, as the more modalities included for processing, the more time it will take to perform the final task and also the more error prone the complete pipeline will be. The results of models trained on data from a single institution and applied on data of other independent institutions (i.e,. TJU and MDA) yield a consistently comparable performance between the proposed modality-agnostic approach and the “T1-T1” and “Multi-4” models. To rigorously validate the proposed modality-agnostic method on diverse multi-institutional data, we chose to first train two different models using i) data from a single institution (UPenn) and ii) multi-institutional data (UPenn + TJU + MDA), and then infer them on multi-institutional diverse data from TCIA, i.e., from institutions not included in the training cohort. The results (Fig. 10) indicate that introducing knowledge from multiple institutions improves the performance of the trained models. Moreover, introducing a brain shape prior through the modality agnostic approach can handle any modality scan improving the performance (Fig. 10) and increasing the applicability of the method. Thus, the benefit of not requiring a specific modality (for the aforementioned reasons) enables us to appraise greater preference for the modality-agnostic training process compared to the “T1-T1”.

**Fig. 11.**
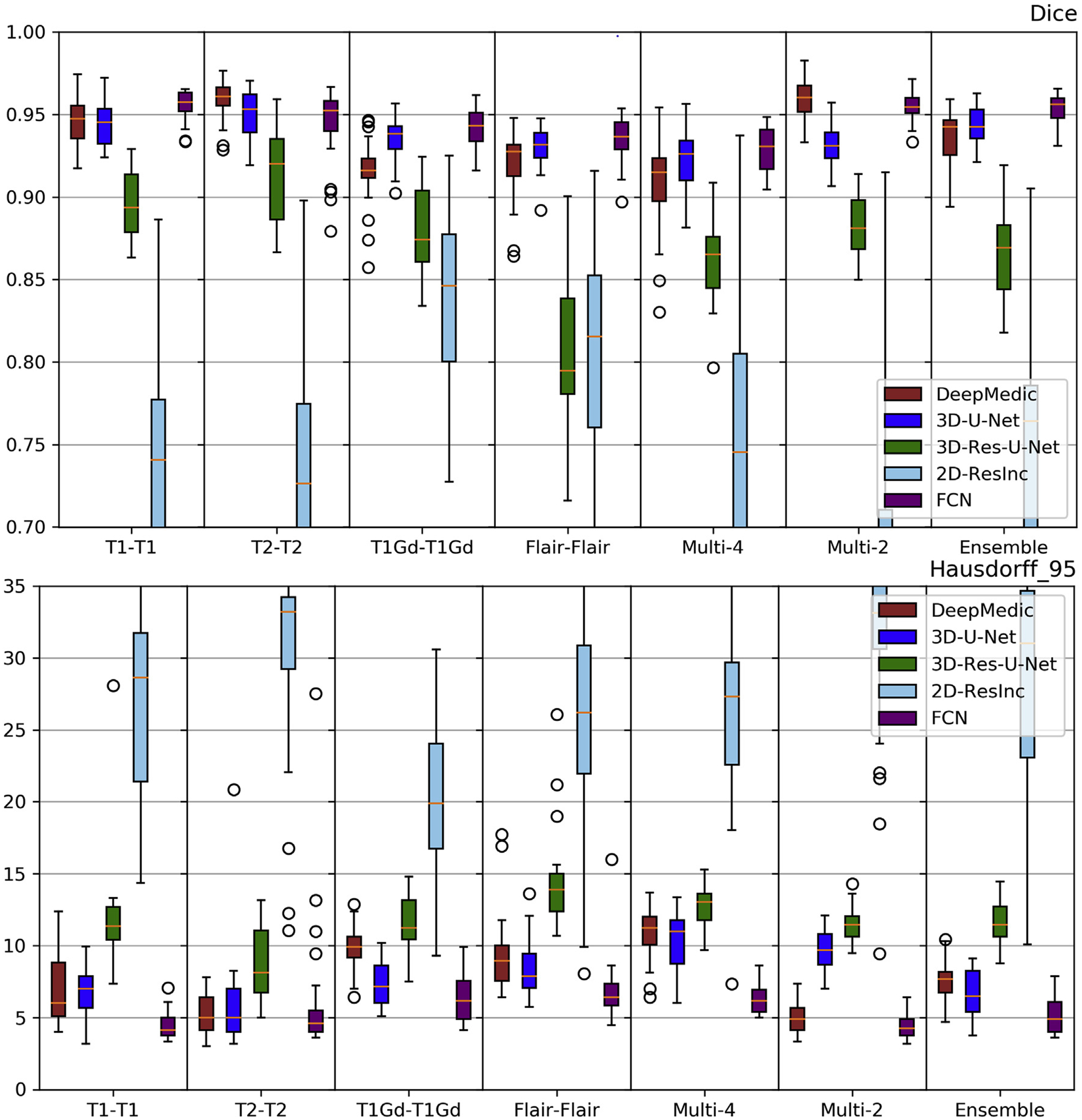
Quantitative (average *Dice* and *Hausdorf f*) evaluation of the various DL network architectures tested on unseen defaced data from an independent institution (WashU). The evaluated models in this figure include training on individual modalities of the UPenn dataset and their ensemble using majority voting, as well as multi-modality training.

**Fig. 12.**
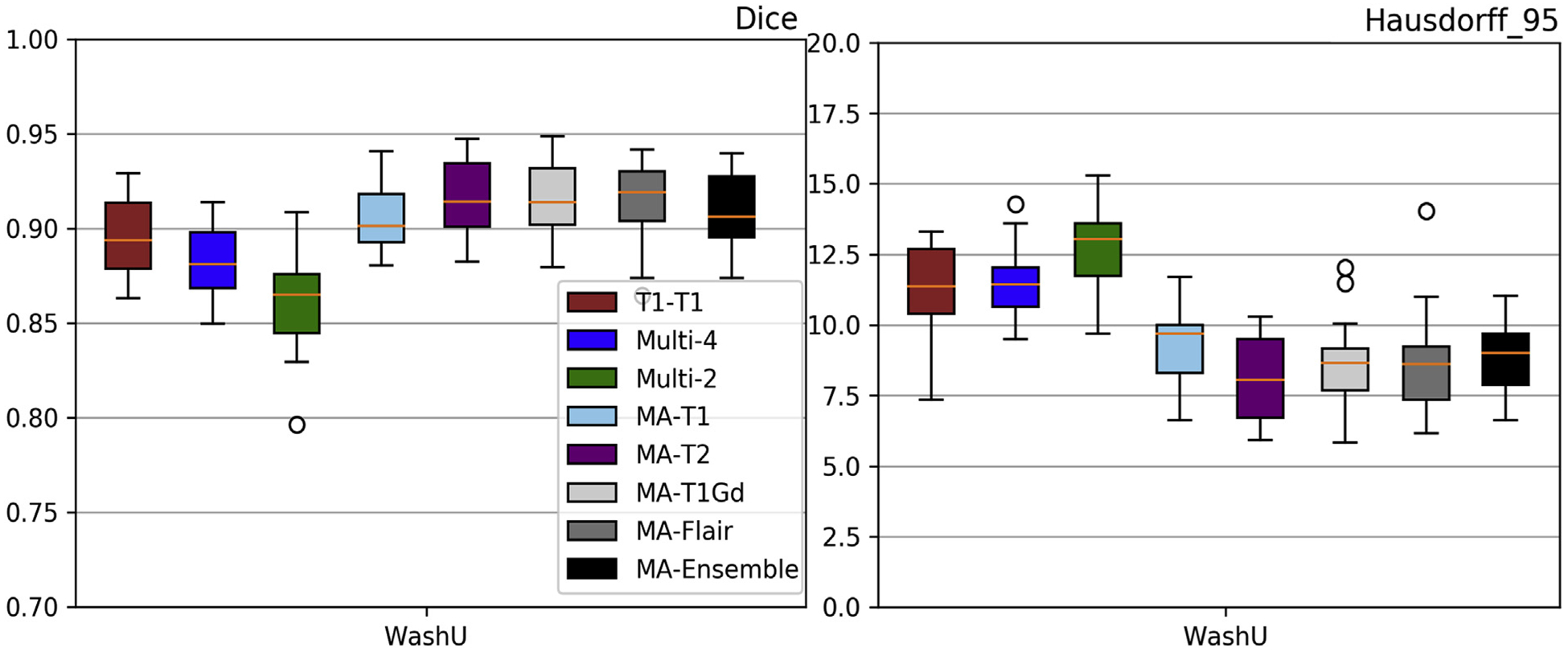
Evaluation results of the selected 3D-Res-U-Net on the Modality-Agnostic training process tested on unseen defaced data from an independent institution (WashU). Average *Dice* and *Hausdorf f*_95_ metrics are shown in the left and right columns, respectively. Results also include the “T1-T1” and the “Multi-4” models for comparison purposes.

While aiming on a practical brain extraction tool, generalizable across multiple institutions and multiple modalities, to facilitate the current paradigm for multi-institutional collaborations through data sharing, the current study has three major limitations: a) did not use any defaced/semi-skull-stripped data, b) consideration of only pre-operative scans, and c) consideration of only brain glioblastomas. Specifically, our models were trained on images prior to any defacing, which is a common practice mandated by many institutions for data sharing purposes, in order to comply with privacy regulations. We therefore identified another independent retrospective cohort of 120 scans from Washington University in St. Louis (WashU) that have followed the common practice of data sharing after defacing. We attempted to evaluate our current models on defaced data and we noted an inferior performance (Figs. 11–12) when compared to the existing results. However, our modality agnostic approach shows promise for a potential solution that can be used as a harmonized pre-processing step giving rise to various paradigm shift approaches for multi-institutional collaborations, such as incremental learning (Li and Hoiem) and federated learning (Sheller et al., 1810). We think that the intrinsic shape prior is what makes the modality agnostic performance being more robust to missing skull. Second, the current study evaluated all models on pre-operative pathologies, and did not consider any post-resection tumor scans. Although the transition from pre-operative to post-operative scans might seem straightforward, there may be issues introduced due to the presence of cavities. Another limitation of our study is the use of subjects with only one type of pathology, i.e., glioblastoma. While the results on one type of pathology may not represent the entire spectrum of brain diseases, we hypothesize that the modality agnostic approach could be effective when on scans with other benign or malignant pathologies, such as traumatic brain injuries, intracranial infections, meningiomas, low grade gliomas, brain metastases, and more.

## 5. Conclusion

Brain extraction is a key component of computer algorithms that are poised to change the practice of clinical neuroradiology. It may facilitate real time automatic brain lesion detection and segmentation for clinical interpretation. It is therefore critical that this component be not only fast, but also have the flexibility to accommodate different MRI protocols, even with a limited number of sequences, and different hardware platforms. Our study demonstrates that DL brain extraction algorithms have the potential to satisfy those requirements.

In this study, using extensive validation protocols, we have shown that DL methods generally out-perform conventional brain extraction methods, such as BET, and FreeSurfer. Though accuracy is important, practical constraints dictate that the importance of the trained model’s generalizability. Towards this end we introduced a modality-agnostic training method rather than one that needs a specific set or combination of modalities, which forces the model to learn the spatial relationships between the structures in the brain and its shape, as opposed to texture, and thereby overriding the need for a particular modality. In addition, we have also proved that such a model will have comparable (and in most cases better) accuracy to other DL methods while keeping minimal computational and logistical requirements. Finally, we have publicly released the accompanying source code at: https://github.com/CBICA/BrainMaGe.

## Supplementary Material

1

## Figures and Tables

**Fig. 13.**
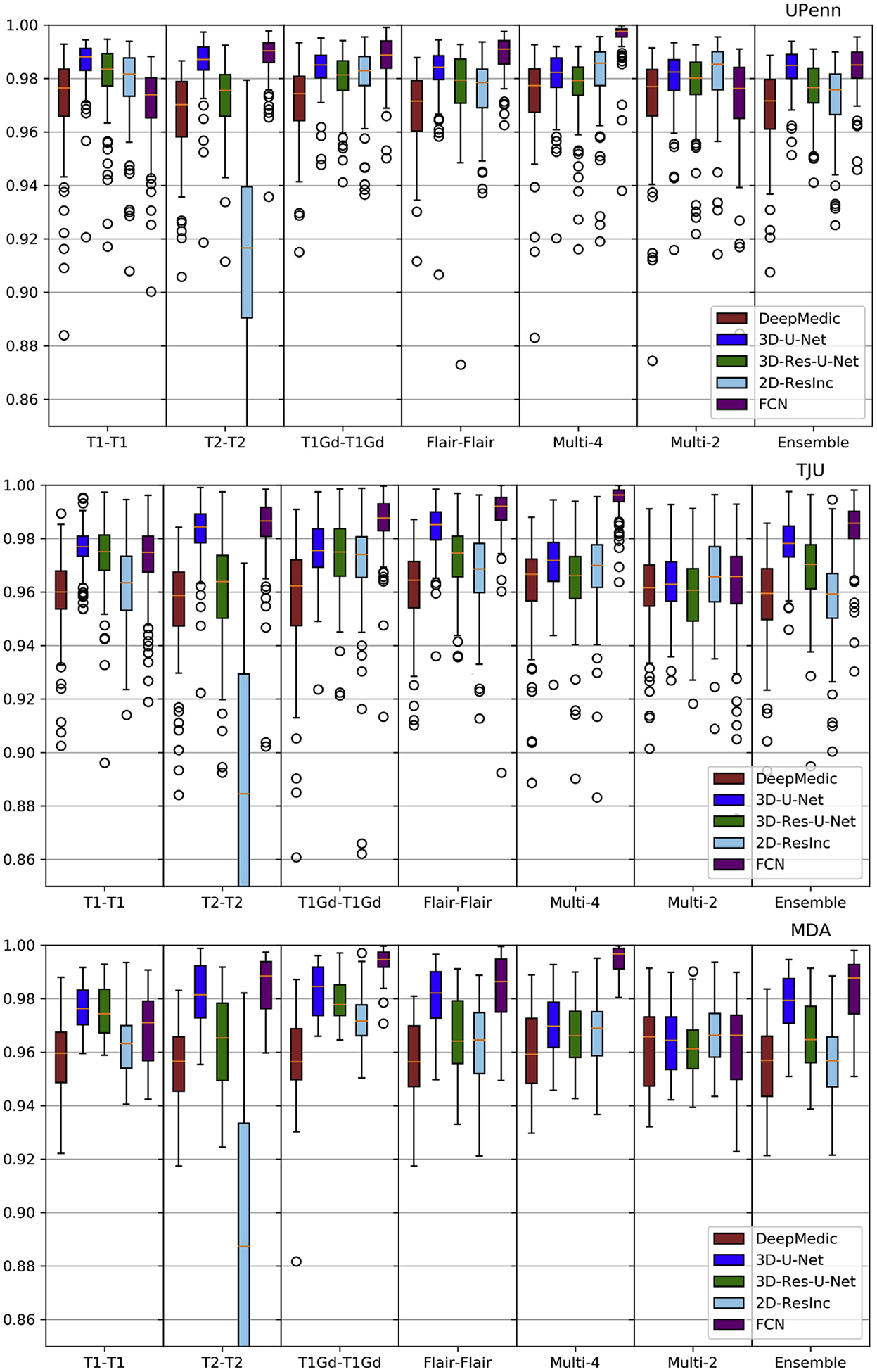
Quantitative (average *Sensitivity*) evaluation of various DL network architectures. From top to bottom rows we see results on the data from (*a*) UPenn, (*b*) TJU, and (*c*) MDA. The evaluated models in this figure include training on individual modalities and their ensemble using majority voting, as well as multi-modality training.

**Fig. 14.**
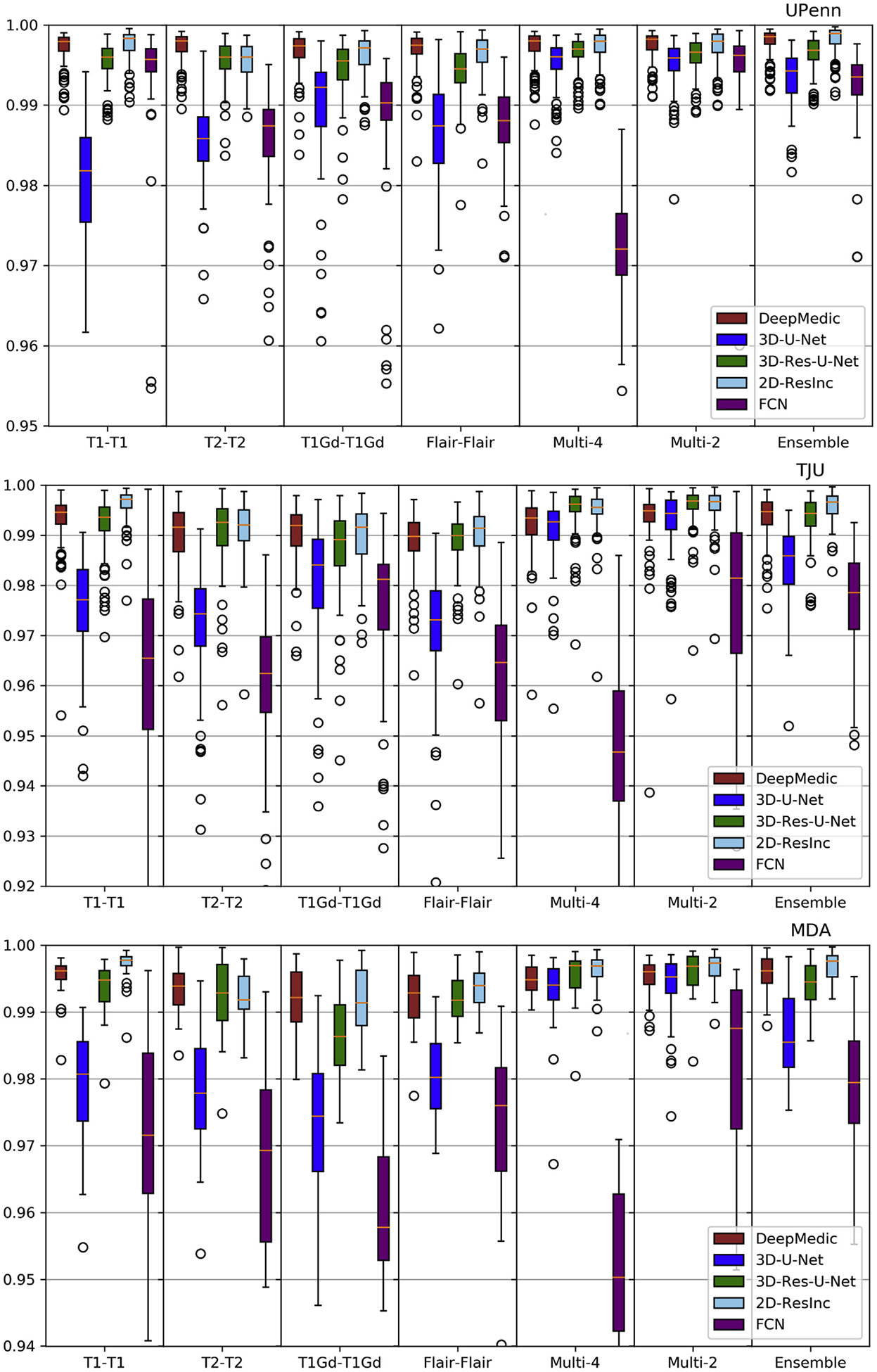
Quantitative (average *Specificity*) evaluation of various DL network architectures. From top to bottom rows we see results on the data from (*a*) UPenn, (*b*) TJU, and (*c*) MDA. The evaluated models in this figure include training on individual modalities and their ensemble using majority voting, as well as multi-modality training.

**Fig. 15.**
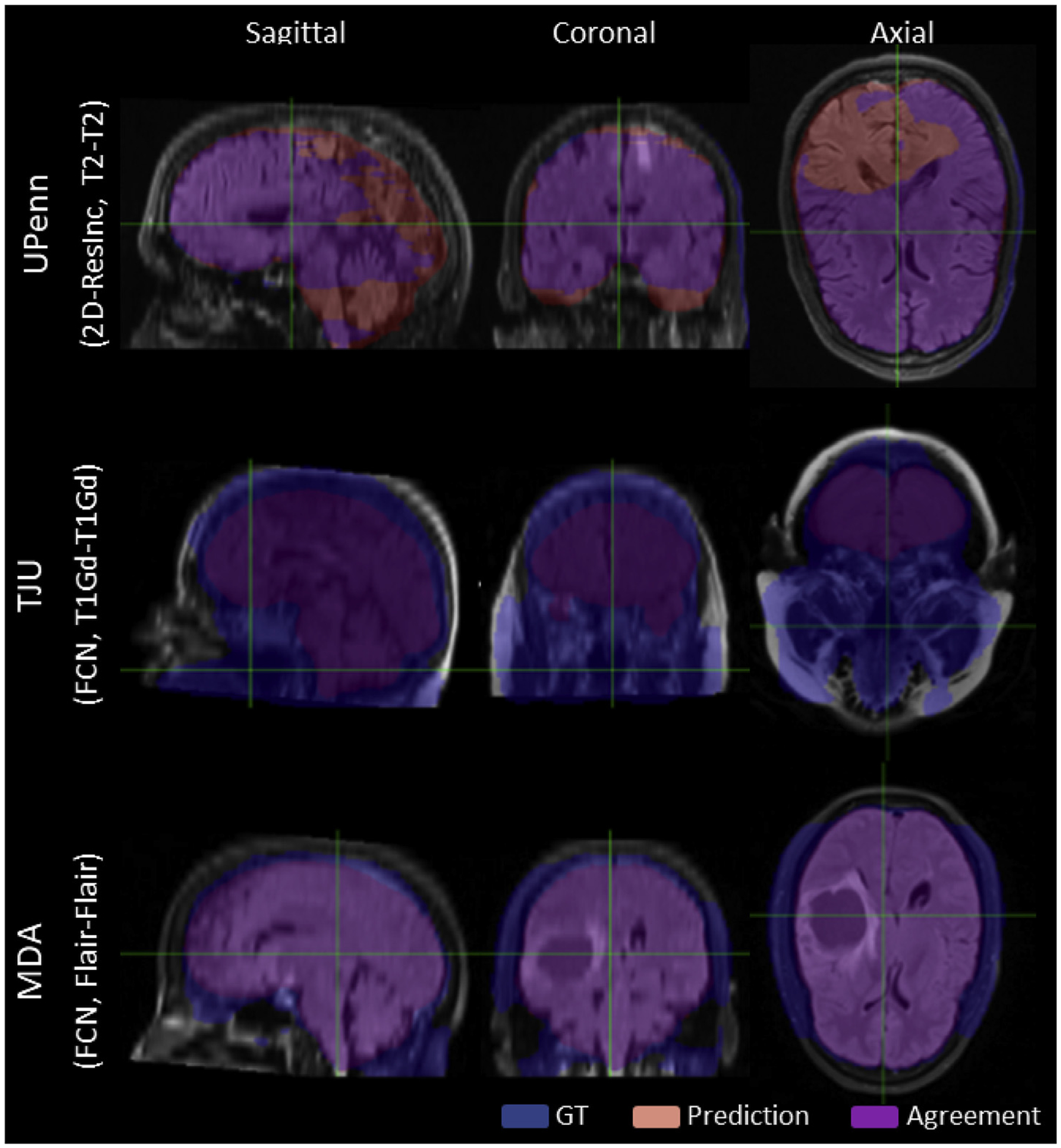
Qualitative Poor Segmentation examples, randomly chosen from each institutions, across all algorithms and after setting a *Thr*_*Dice*_ < 80%.)

**Fig. 16.**
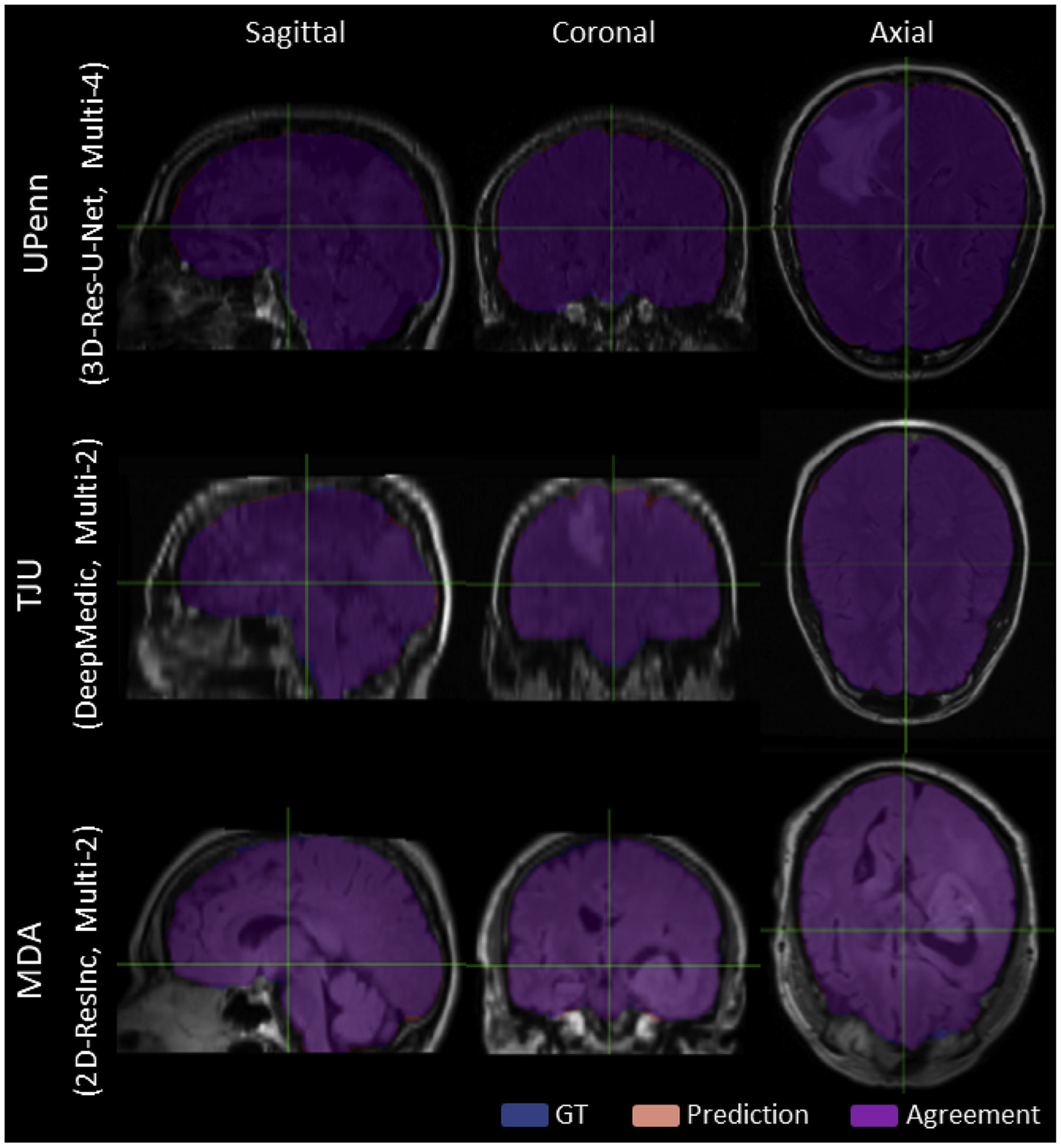
Qualitative Good Segmentation examples, randomly chosen from each institutions, across all algorithms and after setting a *Thr*_*Dice*_ > 98%.)

**Table 1:** Summary of data included in our comparative evaluations.

Dataset	# Subjects.	# mpMRI Scans
UPenn	453	1,812
TJU	152	608
MDA	25	100
TCGA-GBM	82	328
TCGA-LGG	93	372
**Total**	**805**	**3220**

**Table 2:** Time to converge during training. The reported time is in hours and in parenthesis is the number of epochs that each model was trained for before application for inference.

	DeepMedic	3D-U-Net	3D-Res-U-Net	2D-ResInc	FCN
T1-T1	18 (35)	6 (25)	6 (25)	150.5 (96)	3 (25)
T2-T2	26 (35)	6 (25)	6 (25)	71.5 (38)	3 (25)
T1Gd-T1Gd	24 (35)	6 (25)	6 (25)	151.7 (100)	3 (25)
Flair-Flair	18 (35)	6 (25)	6 (25)	147.7 (94)	3 (25)
Multi-2	36 (35)	8 (25)	8 (25)	157.3 (91)	4 (25)
Multi-4	45 (35)	8 (25)	8 (25)	113.6 (56)	4 (25)
M-A	62 (45)	25 (25)	25 (25)	321.8 (54)	17 (25)

## Footnotes

2 https://cbica.github.io/CaPTk/preprocessing_brats.html.

3 https://github.com/deepmedic/deepmedic.

4 v.0.7.1 [commit dbdc1f1], code.

## References

[R1] Bakas S, Reyes M, Jakab A, Bauer S, Rempfler M, Crimi A, Shinohara RT, Berger C, Ha SM, Rozycki M, Prastawa M, Alberts E, Lipková J, Freymann JB, Kirby JS, Bilello M, Fathallah-Shaykh HM, Wiest R, Kirschke J, Wiestler B, Colen RR, Kotrotsou A, LaMontagne P, Marcus DS, Milchenko M, Nazeri A, Weber M, Mahajan A, Baid U, Kwon D, Agarwal M, Alam M, Albiol A, Albiol A, Varghese A, Tuan TA, Arbel T, Avery A, Pranjal B, Banerjee S, Batchelder T, Batmanghelich KN, Battistella E, Bendszus M, Benson E, Bernal J, Biros G, Cabezas M, Chandra S, Chang Y, Identifying the best machine learning algorithms for brain tumor segmentation, progression assessment, and overall survival prediction in the BRATS challenge, CoRR abs/1811.02629. arXiv:1811.02629. http://arxiv.org/abs/1811.02629.

[R2] Bakas S, Akbari H, Sotiras A, Bilello M, Rozycki M, Kirby JS, Freymann JB, Farahani K, Davatzikos C, 2017 Advancing the cancer genome atlas glioma mri collections with expert segmentation labels and radiomic features. Nat. Sci. Data 4, 170117 10.1038/sdata.2017.117. URL. PMC5685212 28872634

[R3] Bakas S, Akbari H, Sotiras A, Bilello M, Rozycki M, Kirby J, Freymann J, Farahani K, Davatzikos C, 2017 Segmentation Labels and Radiomic Features for the Pre-operative Scans of the Tcga-Gbm Collection. 10.7937/K9/TCIA.2017.KLXWJJ1Q. PMC5685212 28872634

[R4] Bakas S, Akbari H, Sotiras A, Bilello M, Rozycki M, Kirby J, Freymann J, Farahani K, Davatzikos C, 2017 Segmentation Labels and Radiomic Features for the Pre-operative Scans of the Tcga-Lgg Collection. 10.7937/K9/TCIA.2017.GJQ7R0EF. PMC5685212 28872634

[R5] Çiçek Ö, Abdulkadir A, Lienkamp SS, Brox T, Ronneberger O, 2016 3D U-Net: Learning Dense Volumetric Segmentation from Sparse Annotation In: Ourselin S, Joskowicz L, Sabuncu M, Unal G, Wells W (Eds.), Medical Image Computing and Computer-Assisted Intervention – MICCAI 2016. MICCAI 2016. Lecture Notes in Computer Science, vol. 9901 Springer, Cham 10.1007/978-3-319-46723-8_49.

[R6] Clark K, Vendt B, Smith K, Freymann J, Kirby J, Koppel P, Moore S, Phillips S, Maffitt D, Pringle M, Tarbox L, Prior F, 2013 The cancer imaging archive (tcia): maintaining and operating a public information repository. J. Digit. Imag 26 (6), 1045–1057. 10.1007/s10278-013-9622-7. URL: 10.1007/s10278-013-9622-7. PMC3824915 23884657

[R7] Cox R, Ashburner J, Breman H, Fissell K, Haselgrove C, Holmes C, Lancaster J, Rex D, Smith S, Woodward J, , 2004 A (Sort of) New Image Data Format Standard: Nifti-1: We 150. Neuroimage 22.

[R8] Dale AM, Fischl B, Sereno MI, 1999 Cortical surface-based analysis: I. segmentation and surface reconstruction. Neuroimage 9 (2), 179–194. 10.1006/nimg.1998.0395. URL. http://www.sciencedirect.com/science/article/pii/S1053811998903950. 9931268

[R9] Doshi J, Erus G, Ou Y, Gaonkar B, Davatzikos C, 2013 Multi-atlas skull-stripping. Acad. Radiol 20 (12), 1566–1576. 10.1016/j.acra.2013.09.010. URL. http://www.sciencedirect.com/science/article/pii/S1076633213004182. 24200484 PMC3880117

[R10] Doshi J, Erus G, Habes M, Davatzikos C, 2019 DeepMRSeg: A Convolutional Deep Neural Network for Anatomy and Abnormality Segmentation on MR Images, arXiv E-Prints arXiv:1907.02110arXiv:1907.02110.

[R11] Drozdzal M, Vorontsov E, Chartrand G, Kadoury S, Pal C, 2016 The Importance of Skip Connections in Biomedical Image Segmentation In: Carneiro G, (Eds.), Deep Learning and Data Labeling for Medical Applications. DLMIA 2016, LABELS 2016. Lecture Notes in Computer Science, vol. 10008 Springer, Cham 10.1007/978-3-319-46976-8_19.

[R12] Eskildsen SF, Coupé P, Fonov V, Manjón JV, Leung KK, Guizard N, Wassef SN, Østergaard LR, Collins DL, 2012 Beast: brain extraction based on nonlocal segmentation technique. Neuroimage 59 (3), 2362–2373. 10.1016/j.neuroimage.2011.09.012. URL. http://www.sciencedirect.com/science/article/pii/S1053811911010573. 21945694

[R13] Frisoni GB, Fox NC, Jack CR Jr., Scheltens P, Thompson PM, 2010 The clinical use of structural mri in alzheimer disease. Nat. Rev. Neurol 6, 67 10.1038/nrneurol.2009.215. EP –, review Article. URL. 20139996 PMC2938772

[R14] Geirhos R, Rubisch P, Michaelis C, Bethge M, Wichmann FA, Brendel W. Imagenet-trained cnns are biased towards texture; increasing shape bias improves accuracy and robustness, CoRR abs/1811, 12231. arXiv:1811.12231. URL. http://arxiv.org/abs/1811.12231.

[R15] Gitler AD, Dhillon P, Shorter J, 2017 Neurodegenerative disease: models, mechanisms, and a new hope. Dis. Models Mech 10 (5), 499–502. 10.1242/dmm.030205, 28468935[pmid]. https://www.ncbi.nlm.nih.gov/pubmed/28468935. PMC5451177 28468935

[R16] Haidar H, Soul JS, 2006 Measurement of cortical thickness in 3d brain mri data: validation of the laplacian method. J. Neuroimaging 16 (2), 146–153. 10.1111/j.1552-6569.2006.00036.x. 16629737

[R17] He K, Zhang X, Ren S, Sun J, 2016 Deep residual learning for image recognition. In: Proceedings of the IEEE conference on computer vision and pattern recognition, pp. 770–778.

[R18] Hwang H, Rehman HZU, Lee S, 2019 3d u-net for skull stripping in brain mri. Appl.Sci 9 (3), 569.

[R19] Iglesias JE, Liu C, Thompson PM, Tu Z, 2011 Robust brain extraction across datasets and comparison with publicly available methods. IEEE Trans. Med. Imag 30 (9), 1617–1634. 10.1109/TMI.2011.2138152. 21880566

[R20] Ioffe S, Szegedy C, 2015, 6 Batch Normalization: Accelerating Deep Network Training by Reducing Internal Covariate Shift. In: International Conference on Machine Learning, pp. 448–456.

[R21] Isensee Fabian, Schell Marianne, Pflueger Irada, Brugnara Gianluca, Bonekamp David, Neuberger Ulf, Wick Antje, Schlemmer Heinz-Peter, Heiland Sabine, Wick Wolfgang, Bendszus Martin, Maier-Hein Klaus H., Kickingereder Philipp, 2019 Automated brain extraction of multisequence MRI using artificial neural networks. Hum. Brain Mapp 40 (17), 4952–4964. 10.1002/hbm.24750. 31403237 PMC6865732

[R22] Kamnitsas Konstantinos, Ledig Christian, Newcombe Virginia F.J., Simpson Joanna P., Kane Andrew D., Menon David K., Rueckert Daniel, Glocker Ben, 2017 Efficient multi-scale 3D CNN with fully connected CRF for accurate brain lesion segmentation. Med. Image Anal 36, 61–78. 10.1016/j.media.2016.10.004. ISSN 1361–8415. 27865153

[R23] Kim MM, Parmar HA, Aryal MP, Mayo CS, Balter JM, Lawrence TS, Cao Y, 2019 Developing a pipeline for multiparametric mri-guided radiation therapy: initial results from a phase ii clinical trial in newly diagnosed glioblastoma, Tomography (Ann Arbor, Mich, 30854449[pmid]. URL. https://www.ncbi.nlm.nih.gov/pubmed/30854449, 5 (1) 118–126. 10.18383/j.tom.2018.00035 PMC6403045 30854449

[R24] Klein A, Ghosh SS, Avants B, Yeo B, Fischl B, Ardekani B, Gee JC, Mann J, Parsey RV, 2010 Evaluation of volume-based and surface-based brain image registration methods. Neuroimage 51 (1), 214–220. 10.1016/j.neuroimage.2010.01.091. URL. http://www.sciencedirect.com/science/article/pii/S105381191000114X. 20123029 PMC2862732

[R25] Leote J, Nunes RG, Cerqueira L, Loução R, Ferreira HA, 2018 Reconstruction of white matter fibre tracts using diffusion kurtosis tensor imaging at 1.5t: pre-surgical planning in patients with gliomas. Euro. J. Radiol. Open 5, 20–23. 10.1016/j.ejro.2018.01.002. http://www.sciencedirect.com/science/article/pii/S2352047718300029. PMC5926250 29719853

[R26] Li Z, Hoiem D, 1 12 2018 Learning without Forgetting. In: IEEE Transactions on Pattern Analysis and Machine Intelligence, vol. 40, pp. 2935–2947. 10.1109/TPAMI.2017.2773081 no. 12. 29990101

[R27] Litjens G, Kooi T, Bejnordi BE, Setio AAA, Ciompi F, Ghafoorian M, van der Laak JA, van Ginneken B, Sáanchez CI, 2017 A survey on deep learning in medical image analysis. Med. Image Anal 42, 60–88. 10.1016/j.media.2017.07.005. http://www.sciencedirect.com/science/article/pii/S1361841517301135. 28778026

[R28] Long J, Shelhamer E, Darrell T, 2015 Fully convolutional networks for semantic segmentation. In: Proceedings of the IEEE conference on computer vision and pattern recognition, pp. 3431–3440. 10.1109/TPAMI.2016.2572683 27244717

[R29] MacDonald D, Kabani N, Avis D, Evans AC, 2000 Automated 3-d extraction of inner and outer surfaces of cerebral cortex from mri. Neuroimage 12 (3), 340–356. 10.1006/nimg.1999.0534. URL. http://www.sciencedirect.com/science/article/pii/S1053811999905347. 10944416

[R30] Menze BH, Jakab A, Bauer S, Kalpathy-Cramer J, Farahani K, Kirby J, Burren Y, Porz N, Slotboom J, Wiest R, Lanczi L, Gerstner E, Weber M, Arbel T, Avants BB, Ayache N, Buendia P, Collins DL, Cordier N, Corso JJ, Criminisi A, Das T, Delingette H, Demiralp, Durst CR, Dojat M, Doyle S, Festa J, Forbes F, Geremia E, Glocker B, Golland P, Guo X, Hamamci A, Iftekharuddin KM, Jena R, John NM, Konukoglu E, Lashkari D, Mariz JA, Meier R, Pereira S, Precup D, Price SJ, Raviv TR, Reza SMS, Ryan M, Sarikaya D, Schwartz L, Shin H, Shotton J, Silva CA, Sousa N, Subbanna NK, Szekely G, Taylor TJ, Thomas OM, Tustison NJ, Unal G, Vasseur F, Wintermark M, Ye DH, Zhao L, Zhao B, Zikic D, Prastawa M, Reyes M, Van Leemput K, 2015 The multimodal brain tumor image segmentation benchmark (brats). IEEE Trans. Med. Imag 34 (10), 1993–2024. 10.1109/TMI.2014.2377694. PMC4833122 25494501

[R31] Milletari F, Navab N, Ahmadi S, 2016 V-Net: Fully Convolutional Neural Networks for Volumetric Medical Image Segmentation In: 2016 Fourth International Conference on 3D Vision (3DV), Stanford, CA, pp. 565–571. 10.1109/3DV.2016.79.

[R32] Nadkarni TN, Andreoli MJ, Nair VA, Yin P, Young BM, Kundu B, Pankratz J, Radtke A, Holdsworth R, Kuo JS, Field AS, Baskaya MK, Moritz CH, Meyerand ME, Prabhakaran V, 2015 Usage of fmri for pre-surgical planning in brain tumor and vascular lesion patients: task and statistical threshold effects on language lateralization. Neuroimage: Clin. 7, 415–423. 10.1016/j.nicl.2014.12.014. http://www.sciencedirect.com/science/article/pii/S2213158214001995. 25685705 PMC4310930

[R33] Pedano N, Flanders A, Scarpace L, Mikkelsen T, Eschbacher J, Hermes B, , 2016 Radiology data from the cancer genome atlas low grade glioma [tcga-lgg] collection. Canc. Imag. Archiv 10.7937/K9/TCIA.L4LTD3TK.

[R34] Radue E-W, Barkhof F, Kappos L, Sprenger T, Häaring DA, de Vera A, von Rosenstiel P, Bright JR, Francis G, Cohen JA, 2015 Correlation between brain volume loss and clinical and mri outcomes in multiple sclerosis. Neurology 84 (8), 784–793. 10.1212/WNL.0000000000001281 arXiv:https://n.neurology.org/content/84/8/784.full.pdf. https://n.neurology.org/content/84/8/784. 25632085 PMC4339126

[R35] Rockafellar RT, Wets RJ-B, 2005 Variational Analysis, vol. 317 Springer Science &Business Media.

[R36] Rohlfing T, Zahr NM, Sullivan EV, Pfefferbaum A, 2010 The sri24 multichannel atlas of normal adult human brain structure. Hum. Brain Mapp 31 (5), 798–819. 10.1002/hbm.20906, 20017133[pmid]. https://www.ncbi.nlm.nih.gov/pubmed/20017133. 20017133 PMC2915788

[R37] Ronneberger O, Fischer P, Brox T, 2015 U-Net: Convolutional Networks for Biomedical Image Segmentation In: Navab N, Hornegger J, Wells W, Frangi A (Eds.), Medical Image Computing and Computer-Assisted Intervention – MICCAI 2015. MICCAI 2015. Lecture Notes in Computer Science, vol. 9351 Springer, Cham.

[R38] Scarpace L, Mikkelsen T, Cha S, Rao S, Tekchandani S, Gutman D, , 2016 Radiology data from the cancer genome atlas glioblastoma multiforme [tcga-gbm] collection. Canc. Imag. Arch 10.7937/K9/TCIA. RNYFUYE9.

[R39] Segonne F, Dale AM, Busa E, Glessner M, Salat D, Hahn HK, Fischl B, 2004 A hybrid approach to the skull stripping problem in mri. Neuroimage 22 (3), 1060–1075. 10.1016/j.neuroimage.2004.03.032. 15219578

[R40] Shattuck DW, Sandor-Leahy SR, Schaper KA, Rottenberg DA, Leahy RM, 2001 Magnetic resonance image tissue classification using a partial volume model. Neuroimage 13 (5), 856–876. 10.1006/nimg.2000.0730. URL. http://www.sciencedirect.com/science/article/pii/S1053811900907304. 11304082

[R41] Sheller MJ, Reina GA, Edwards B, Martin J, Bakas S, 2019 Multi-institutional Deep Learning Modeling Without Sharing Patient Data: A Feasibility Study on Brain Tumor Segmentation In: Crimi A, Bakas S, Kuijf H, Keyvan F, Reyes M, van Walsum T (Eds.), Brainlesion: Glioma, Multiple Sclerosis, Stroke and Traumatic Brain Injuries. BrainLes 2018. Lecture Notes in Computer Science, vol. 11383 Springer, Cham 10.1007/978-3-030-11723-8_9. PMC6589345 31231720

[R42] Smith SM, 2002 Fast robust automated brain extraction. Hum. Brain Mapp 17 (3), 143–155. 10.1002/hbm.10062. arXiv https://onlinelibrary.wiley.com/doi/pdf/10.1002/hbm.10062. https://onlinelibrary.wiley.com/doi/abs/10.1002/hbm.10062. 12391568 PMC6871816

[R43] Souza R, Lucena O, Garrafa J, Gobbi D, Saluzzi M, Appenzeller S, Rittner L, Frayne R, Lotufo R, 2018 An open, multi-vendor, multi-field-strength brain mr dataset and analysis of publicly available skull stripping methods agreement. Neuroimage 170, 482–494. 10.1016/j.neuroimage.2017.08.021 segmenting the Brain. http://www.sciencedirect.com/science/article/pii/S1053811917306687. 28807870

[R44] Szegedy C, Ioffe S, Vanhoucke V, Alemi AA, 2017 Inception-v4, inception-resnet and the impact of residual connections on learning. Thirty-First AAAI Conference on Artificial Intelligence.

[R45] Tosun D, Rettmann ME, Naiman DQ, Resnick SM, Kraut MA, Prince JL, 2006 Cortical reconstruction using implicit surface evolution: accuracy and precision analysis. Neuroimage 29 (3), 838–852. 10.1016/j.neuroimage.2005.08.061. URL. http://www.sciencedirect.com/science/article/pii/S1053811905006531. 16269250 PMC4587757

[R46] Ulyanov D, Vedaldi A, Lempitsky VS. Instance normalization: the missing ingredient for fast stylization, CoRR abs/1607, 08022. arXiv:1607.08022. URL. http://arxiv.org/abs/1607.08022.

[R47] Winzeck S, Hakim A, McKinley R, Pinto José A.A.D.S.R., Alves V, Silva C, Pisov M, Krivov E, Belyaev M, Monteiro M, Oliveira A, Choi Y, Paik MC, Kwon Y, Lee H, Kim BJ, Won J-H, Islam M, Ren H, Robben D, Suetens P, Gong E, Niu Y, Xu J, Pauly JM, Lucas C, Heinrich MP, Rivera LC, Castillo LS, Daza LA, Beers AL, Arbelaezs P, Maier O, Chang K, Brown JM, Kalpathy-Cramer J, Zaharchuk G, Wiest R, Reyes M, 2018 Isles 2016 and 2017-benchmarking ischemic stroke lesion outcome prediction based on multispectral mri. Front. Neurol 9 10.3389/fneur.2018.00679, 679–679, 30271370 [pmid]. https://www.ncbi.nlm.nih.gov/pubmed/30271370. 30271370 PMC6146088

[R48] Woods RP, Mazziotta JC, Cherry R, Simon, Mri-pet registration with automated algorithm, Journal of Computer Assisted Tomography 17 (4). URL https://journals.lww.com/jcat/Fulltext/1993/07000/MRI_PET_Registration_with_Automated_Algorithm.4.aspx. 10.1097/00004728-199307000-00004 8331222

[R49] Yushkevich PA, Piven J, Hazlett HC, Smith RG, Ho S, Gee JC, Gerig G, 2006 User-guided 3d active contour segmentation of anatomical structures: significantly improved efficiency and reliability. Neuroimage 31 (3), 1116–1128. 16545965 10.1016/j.neuroimage.2006.01.015

[R50] Yushkevich PA, Pluta J, Wang H, Wisse LE, Das S, Wolk D, 2016 Fast automatic segmentation of hippocampal subfields and medial temporal lobe subregions in 3 tesla and 7 tesla t2-weighted mri, Alzheimer’s & Dementia. J. Alzheimer’s Assoc 12 (7), P126–P127.

[R51] Yushkevich PA, Pashchinskiy A, Oguz I, Mohan S, Schmitt JE, Stein JM, Zukić D, Vicory J, McCormick M, Yushkevich N, , 2019 User-guided segmentation of multi-modality medical imaging datasets with itk-snap. Neuroinformatics 17 (1), 83–102. 29946897 10.1007/s12021-018-9385-x PMC6310114

[R52] Zhao L, Ruotsalainen U, Hirvonen J, Hietala J, Tohka J, 2010 Automatic cerebral and cerebellar hemisphere segmentation in 3d mri: adaptive disconnection algorithm. Med. Image Anal 14 (3), 360–372. 10.1016/j.media.2010.02.001. http://www.sciencedirect.com/science/article/pii/S1361841510000162. 20303318

