# Supplementary material for: Brain extraction on MRI scans in presence of diffuse glioma: Multi-institutional performance evaluation of deep learning methods and robust modality-agnostic training
Source: Neuroimage. Author manuscript; Available in PMC 2020 Oct 30. (PMC7597856; doi:10.1016/j.neuroimage.2020.117081)
Supplement: 1 [file NIHMS1639060-supplement-1.pdf]

6. Supplementary Material

Table 3: Median and IQR for the *Dice* metric for all trained models.

Site	Model	BET	FreeSurfer	DeepMedic	3D-U-Net	3D-Res-U-Net	2D-ResInc	FCN
UPenn	T1-T1	.96±.01	.92±.01	.98±.01	.94±.02	.98±.01	.97±.00	.98±.01
	T2-T2			.97±.01	.96±.01	.97±.00	.96±.01	.94±.02
	T1Gd-T1Gd			.97±.01	.97±.01	.97±.01	.97±.00	.98±.01
	Flair-Flair			.97±.01	.96±.01	.97±.00	.96±.01	.98±.00
	Multi-2			.98±.01	.98±.01	.98±.00	.93±.01	.98±.01
	Multi-4			.98±.01	.98±.01	.98±.00	.97±.01	.98±.01
	Ensemble			.98±.01	.97±.01	.98±.00	.97±.00	.98±.01
	MA-T1			.97±.01	.97±.01	.98±.01	.97±.00	.98±.01
	MA-T2			.97±.01	.97±.01	.97±.01	.97±.01	.98±.01
	MA-T1Gd			.97±.01	.97±.00	.98±.01	.97±.01	.98±.01
	MA-Flair			.97±.00	.97±.00	.97±.00	.97±.01	.98±.00
	MA-Ensemble			.97±.01	.98±.00	.98±.00	.97±.01	.98±.01
TJU	T1-T1	.83±.06	.91±.03	.96±.01	.93±.03	.97±.01	.90±.06	.97±.00
	T2-T2			.95±.01	.93±.02	.96±.01	.90±.03	.91±.06
	T1Gd-T1Gd			.95±.01	.94±.02	.96±.01	.94±.03	.96±.01
	Flair-Flair			.95±.01	.92±.03	.96±.01	.91±.04	.96±.01
	Multi-2			.96±.01	.96±.01	.97±.00	.88±.03	.97±.00
	Multi-4			.96±.01	.96±.01	.97±.00	.93±.04	.97±.00
	Ensemble			.96±.01	.95±.02	.97±.01	.94±.02	.96±.00
	MA-T1			.95±.01	.95±.02	.95±.02	.88±.06	.97±.00
	MA-T2			.95±.01	.95±.01	.95±.01	.92±.04	.96±.01
	MA-T1Gd			.95±.02	.96±.01	.96±.01	.95±.03	.96±.01
	MA-Flair			.95±.01	.95±.01	.95±.01	.93±.04	.96±.01
	MA-Ensemble			.96±.01	.96±.01	.96±.01	.94±.03	.96±.01
MDA	T1-T1	.87±.07	.91±.02	.96±.01	.94±.03	.97±.01	.91±.07	.97±.00
	T2-T2			.96±.01	.94±.02	.96±.00	.92±.04	.92±.05
	T1Gd-T1Gd			.96±.01	.92±.03	.95±.01	.90±.02	.96±.01
	Flair-Flair			.95±.01	.94±.01	.96±.00	.93±.02	.96±.01
	Multi-2			.96±.01	.97±.01	.97±.01	.88±.04	.97±.00
	Multi-4			.96±.01	.97±.00	.97±.01	.94±.03	.97±.00
	Ensemble			.96±.01	.95±.01	.97±.00	.94±.02	.97±.01
	MA-T1			.96±.01	.96±.01	.96±.02	.92±.04	.97±.01
	MA-T2			.95±.01	.95±.02	.96±.01	.94±.02	.96±.01
	MA-T1Gd			.95±.01	.95±.02	.95±.02	.90±.05	.96±.01
	MA-Flair			.96±.01	.96±.01	.96±.01	.95±.02	.96±.01
	MA-Ensemble			.96±.01	.96±.01	.96±.01	.95±.02	.97±.01
WashU	T1-T1	.96±.01	.95±.01	.94±.01	.94±.02	.89±.03	.95±.01	.74±.09
	T2-T2			.96±.01	.95±.02	.92±.04	.95±.01	.72±.09
	T1Gd-T1Gd			.91±.01	.93±.01	.87±.04	.94±.01	.84±.07
	Flair-Flair			.92±.01	.93±.01	.79±.05	.93±.01	.81±.09
	Multi-2			.91±.02	.92±.02	.86±.03	.93±.02	.74±.12
	Multi-4			.96±.01	.93±.01	.88±.02	.95±.00	.62±.18
	Ensemble			.94±.02	.94±.01	.86±.03	.95±.01	.76±.09
	MA-T1			.93±.01	.92±.02	.90±.02	.94±.01	.84±.06
	MA-T2			.93±.03	.91±.02	.91±.03	.94±.01	.90±.04
	MA-T1Gd			.94±.02	.92±.02	.91±.02	.94±.01	.86±.05
	MA-Flair			.91±.03	.92±.02	.91±.02	.94±.02	.78±.12
	MA-Ensemble			.93±.02	.92±.02	.90±.03	.94±.01	.83±.06

Table 4: Median and IQR for the $Hausdorff$ metric for all trained models.

Site	Model	BET	FreeSurfer	DeepMedic	3D-U-Net	3D-Res-U-Net	2D-ResInc	FCN
UPenn	T1-T1	4.12±1.83	7.34±1.46	2.23±1.41	11.4±2.98	1.73±1.03	2.23±1.0	1.41±1.03
	T2-T2			2.23±1.0	19.2±2.84	2.23±.82	3.60±1.18	19.2±9.91
	T1Gd-T1Gd			2.23±1.26	3.0±2.00	2.0±1.18	2.82±.92	1.73±1.22
	Flair-Flair			2.23±1.0	5.19±5.85	2.23±1.0	3.0±1.55	2.0±1.09
	Multi-2			2.0±1.41	1.73±1.41	1.73±1.03	5.91±.92	1.41±1.23
	Multi-4			2.23±1.41	2.0±1.49	1.73±1.03	2.23±1.0	1.41±1.03
	Ensemble			2.23±1.26	2.23±1.13	2.0±.92	2.23±.82	2.0±.92
	MA-T1			2.44±1.0	2.23±1.04	2.0±1.03	2.23±1.0	1.73±1.03
	MA-T2			2.23±1.15	2.23±1.0	2.23±1.0	2.82±1.22	2.0±1.03
	MA-T1Gd			2.44±1.67	2.23±1.0	2.0±1.09	2.44±1.0	1.73±1.22
	MA-Flair			2.44±1.00	2.23±.82	2.23±.82	2.82±1.63	2.0±.71
	MA-Ensemble			2.23±1.0	2.0±.61	1.73±1.03	2.23±.76	1.41±.82
TJU	T1-T1	20.6±4.44	11.3±8.40	4.13±2.85	11.6±4.08	3.08±1.58	14.2±6.92	3.0±.71
	T2-T2			4.58±1.78	14.7±7.25	4.0±1.83	10.2±3.86	21.4±15.9
	T1Gd-T1Gd			4.58±2.36	5.83±5.57	4.12±2.76	5.83±5.49	3.60±2.19
	Flair-Flair			4.89±2.04	9.05±4.33	4.12±1.68	13.0±8.90	4.12±1.67
	Multi-2			4.12±2.22	4.0±2.76	2.82±.92	12.0±5.61	3.0±.71
	Multi-4			4.0±2.0	3.39±2.19	3.0±.86	9.40±7.14	3.0±.71
	Ensemble			3.60±1.47	5.38±2.87	3.0±1.35	7.07±3.07	3.16±.91
	MA-T1			5.56±2.85	5.29±3.0	4.63±2.34	16.6±11.5	3.0±1.55
	MA-T2			5.65±2.17	5.0±2.29	4.89±2.25	10.7±6.36	4.0±1.76
	MA-T1Gd			5.38±4.62	4.18±3.08	4.0±2.38	5.0±8.71	3.60±2.09
	MA-Flair			4.69±2.08	4.69±2.10	4.69±1.83	8.24±5.71	3.87±1.83
	MA-Ensemble			4.0±1.86	4.24±2.37	4.12±2.09	7.14±5.57	3.16±1.29
MDA	T1-T1	11.3±8.40	9.69±8.26	3.60±1.47	10.2±5.31	2.44±1.22	13.6±7.62	2.82±.92
	T2-T2			4.12±2.09	16.2±6.66	3.60±1.30	9.27±4.59	20.1±13.8
	T1Gd-T1Gd			4.24±2.34	10.8±6.07	4.58±2.83	14.8±4.93	3.60±1.69
	Flair-Flair			4.58±3.39	7.07±3.10	3.74±1.19	9.0±5.67	3.31±1.47
	Multi-2			3.60±2.09	3.0±2.02	2.23±1.08	11.4±4.49	2.82±.71
	Multi-4			3.31±2.27	3.0±1.88	2.44±1.76	8.06±7.14	2.82±1.08
	Ensemble			3.16±1.35	4.58±3.18	3.16±1.29	7.0±3.94	3.0±1.29
	MA-T1			4.47±2.22	4.12±3.00	3.74±2.38	11.8±7.78	2.82±1.08
	MA-T2			4.58±2.16	4.12±2.42	3.60±1.77	7.34±5.24	3.31±1.41
	MA-T1Gd			6.48±4.01	5.09±3.10	4.58±2.05	15.3±8.25	3.46±1.41
	MA-Flair			4.69±2.16	3.60±2.09	3.74±2.0	5.09±3.25	3.16±1.64
	MA-Ensemble			3.74±2.0	3.60±2.38	3.46±1.86	6.0±4.86	3.0±1.76
WashU	T1-T1	3.60±.96	4.58±.85	6.0±3.73	7.0±2.21	11.3±2.29	4.12±1.25	28.6±10.3
	T2-T2			5.0±2.28	5.0±3.0	8.12±4.33	4.58±1.47	33.1±4.99
	T1Gd-T1Gd			9.89±1.46	7.14±2.60	11.2±2.71	6.16±2.65	19.8±7.30
	Flair-Flair			8.94±2.45	7.87±2.36	13.8±2.63	6.40±1.51	26.1±8.91
	Multi-2			11.2±1.95	11.0±3.02	13.0±1.85	6.16±1.54	27.3±7.11
	Multi-4			4.89±1.53	9.69±2.15	11.4±1.41	4.24±1.15	33.1±4.95
	Ensemble			7.68±1.47	6.48±2.86	11.4±2.09	4.89±2.08	31.0±11.6
	MA-T1			8.12±1.32	8.36±1.56	9.69±1.69	5.38±2.05	18.9±10.5
	MA-T2			7.61±2.14	9.43±1.68	8.06±2.77	5.38±2.10	15.1±8.44
	MA-T1Gd			7.07±1.54	7.81±2.87	8.66±1.48	5.38±1.33	18.7±8.99
	MA-Flair			9.48±2.43	8.06±1.77	8.60±1.87	5.38±2.62	24.7±9.06
	MA-Ensemble			8.54±1.0	8.60±1.48	9.0±1.82	5.74±2.10	19.7±9.04

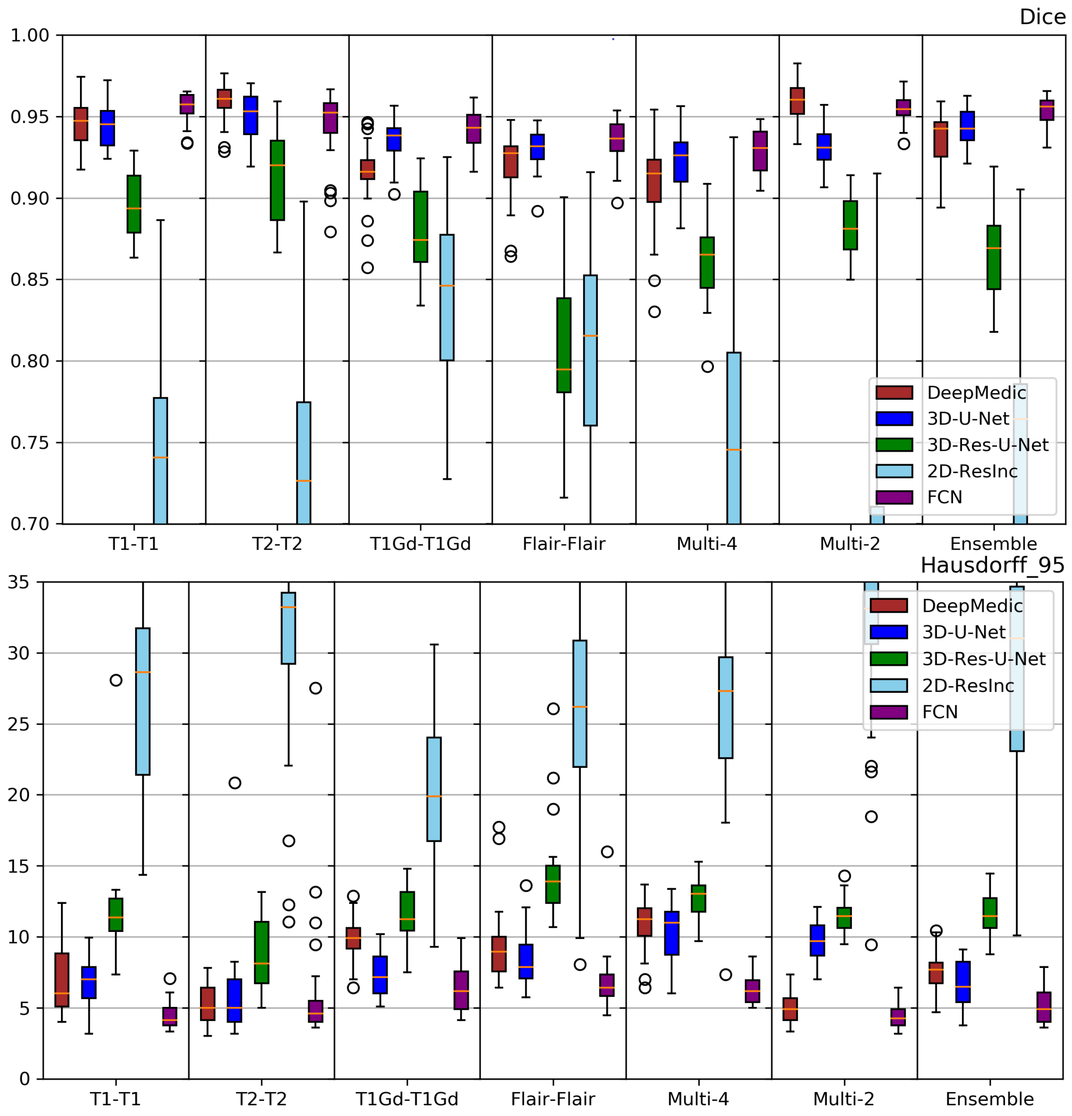

Figure 11: Quantitative (average *Dice* and *Hausdorff*) evaluation of the various DL network architectures tested on unseen defaced data from an independent institution (WashU). The evaluated models in this figure include training on individual modalities of the UPenn dataset and their ensemble using majority voting, as well as multi-modality training.

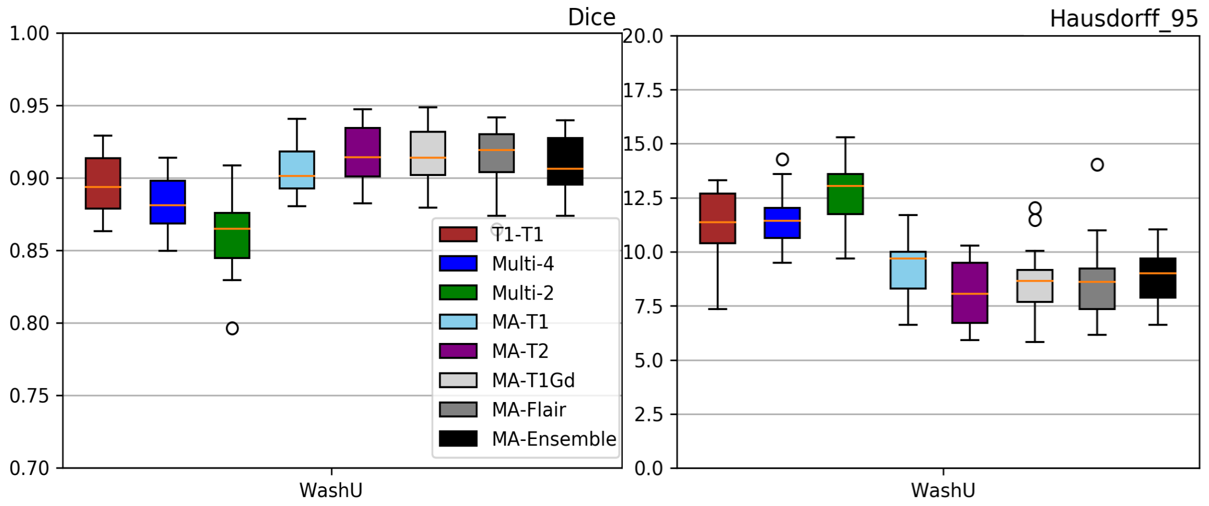

Figure 12: Evaluation results of the selected 3D-Res-U-Net on the Modality-Agnostic training process tested on unseen defaced data from an independent institution (WashU). Average *Dice* and $Hausdorff_{95}$ metrics are shown in the left and right columns, respectively. Results also include the "T1-T1" and the "Multi-4" models for comparison purposes.

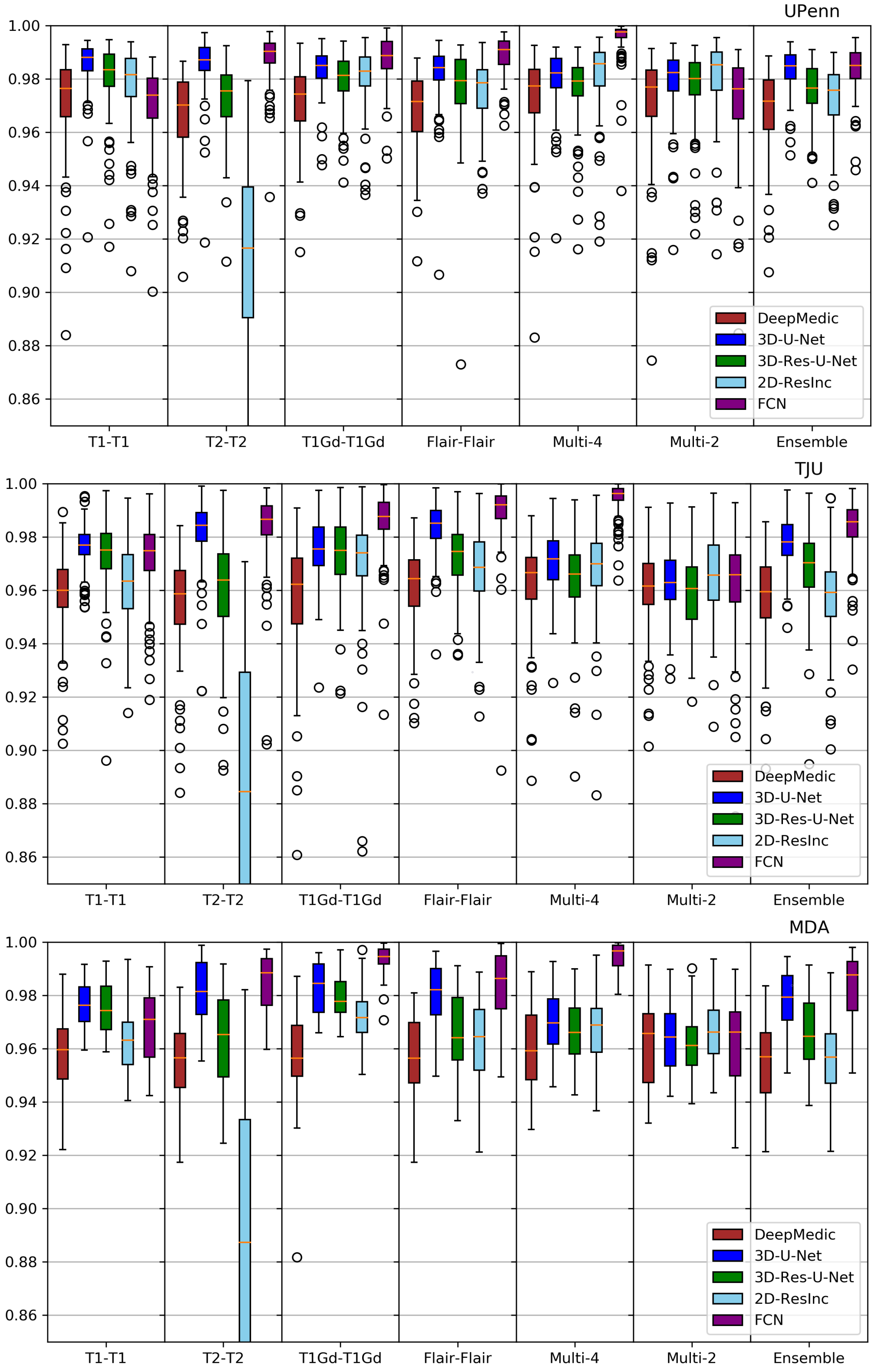

Figure 13: Quantitative (average *Sensitivity*) evaluation of various DL network architectures. From top to bottom rows we see results on the data from (*a*) UPenn, (*b*) TJU, and (*c*) MDA. The evaluated models in this figure include training on individual modalities and their ensemble using majority voting, as well as multi-modality training.

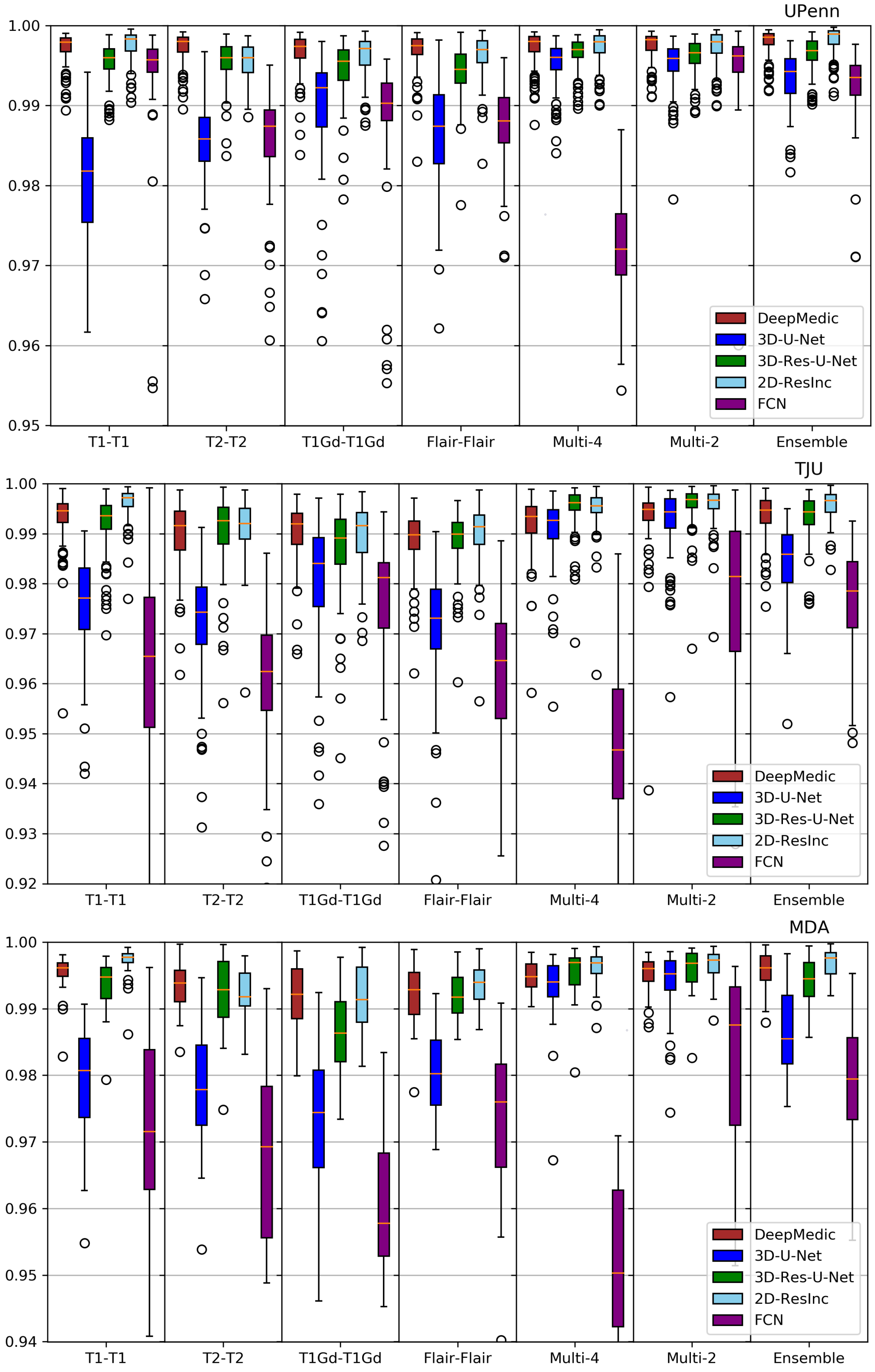

Figure 14: Quantitative (average $Specificity$) evaluation of various DL network architectures. From top to bottom rows we see results on the data from (a) UPenn, (b) TJU, and (c) MDA. The evaluated models in this figure include training on individual modalities and their ensemble using majority voting, as well as multi-modality training.

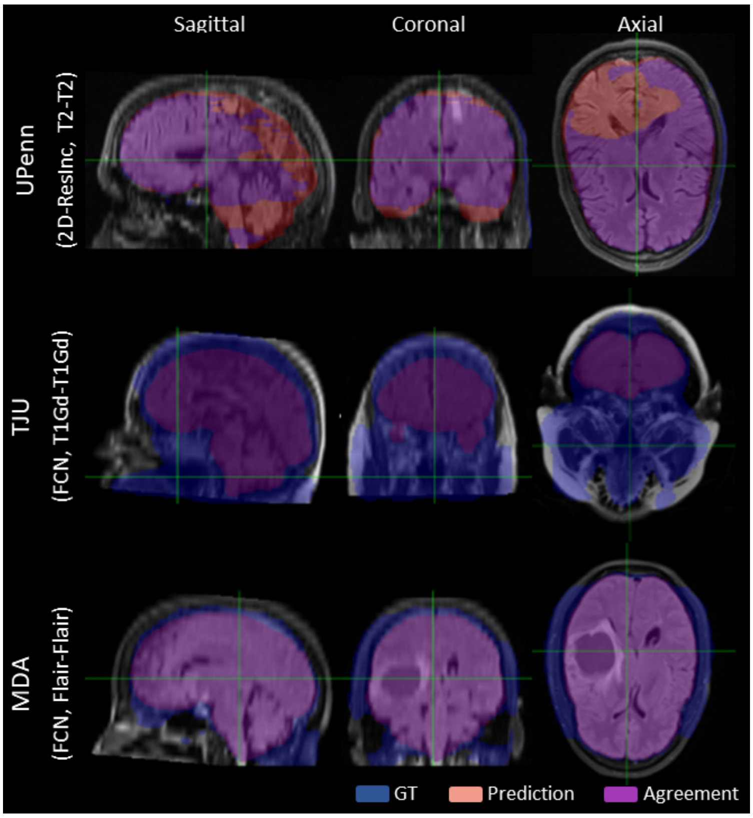

Figure 15: Qualitative Poor Segmentation examples, randomly chosen from each institutions, across all algorithms and after setting a $Thr_{Dice} <$ 80%.)

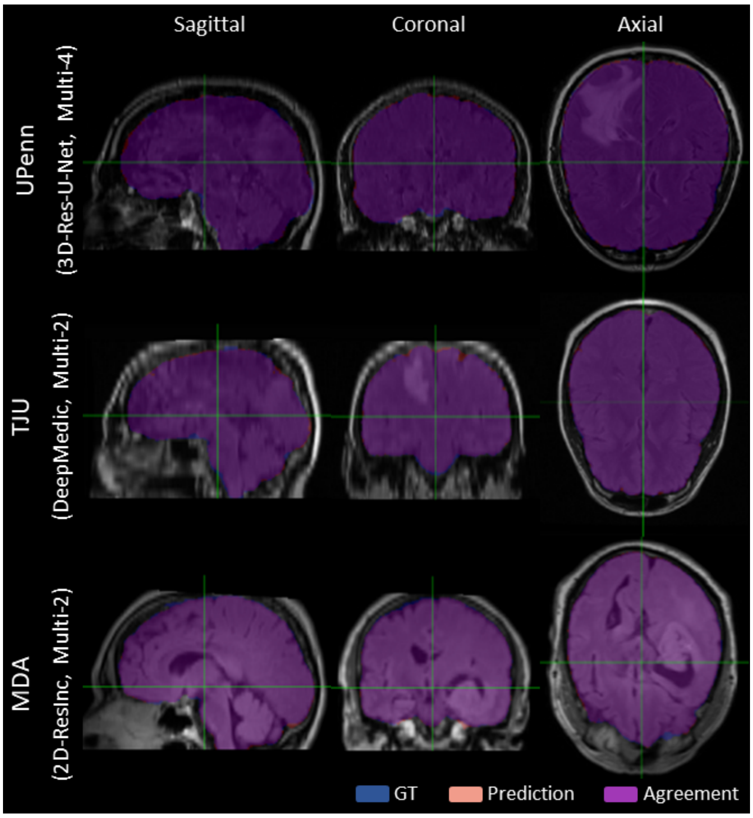

Figure 16: Qualitative Good Segmentation examples, randomly chosen from each institutions, across all algorithms and after setting a Thr_{Dice} > 98%.)
